# FOXC1 promotes HCC proliferation and metastasis by Upregulating DNMT3B to induce DNA Hypermethylation of CTH promoter

**DOI:** 10.1186/s13046-021-01829-6

**Published:** 2021-02-01

**Authors:** Zhuoying Lin, Wenjie Huang, Qin He, Dongxiao Li, Zhihui Wang, Yangyang Feng, Danfei Liu, Tongyue Zhang, Yijun Wang, Meng Xie, Xiaoyu Ji, Mengyu Sun, Dean Tian, Limin Xia

**Affiliations:** 1 Department of Gastroenterology, Institute of Liver and Gastrointestinal Diseases, Hubei Key Laboratory of Hepato-Pancreato-Biliary Diseases, Tongji Hospital of Tongji Medical College, Huazhong University of Science and Technology, Wuhan, 430030 Hubei Province China; 2 Hepatic Surgery Center, Tongji Hospital, Tongji Medical College, Huazhong University of Science and Technology; Clinical Medicine Research Center for Hepatic Surgery of Hubei Province, Key Laboratory of Organ Transplantation, Ministry of Education and Ministry of Public Health, Wuhan, 430030 Hubei China

**Keywords:** Forkhead box C1, Cysteine metabolism, Reactive oxygen species, Cystathionine γ-lyase, DNA methylation

## Abstract

**Background:**

Forkhead box C1 (FOXC1), as a member of the FOX family, is important for promote HCC invasion and metastasis. FOX family protein lays a pivotal role in metabolism. ROS is involved in tumor progression and is associated with the expression of lots of transcription factors. We next explored the mechanism underlying FOXC1 modulating the metabolism and ROS hemostasis in HCC.

**Methods:**

We used amino acids arrays to verify which metabolism is involved in FOXC1-induced HCC. The kits were used to detect the ROS levels in HCC cells with over-expression or down-expression of FOXC1. After identified the downstream target genes and candidate pathway which regulated by FOXC1 during HCC progression in vitro and in vivo, we used western blot, immunohistochemistry, bisulfite genomic sequencing, methylation-specific PCR, chromatin immunoprecipitation analysis and luciferase reporter assays to explore the relationship of FOXC1 and downstream genes. Moreover, the correlation between FOXC1 and target genes and the correlation between target genes and the recurrence and overall survival were analyzed in two independent human HCC cohorts.

**Results:**

Here, we reported that FOXC1 could inhibit the cysteine metabolism and increase reactive oxygen species (ROS) levels by regulating cysteine metabolism-related genes, cystathionine γ-lyase (CTH). Overexpression of CTH significantly suppressed FOXC1-induced HCC proliferation, invasion and metastasis, while the reduction in cell proliferation, invasion and metastasis caused by the inhibition of FOXC1 could be reversed by knockdown of CTH. Meanwhile, FOXC1 upregulated de novo DNA methylase 3B (DNMT3B) expression to induce DNA hypermethylation of *CTH* promoter, which resulted in low expression of CTH in HCC cells. Moreover, low levels of ROS induced by N-acetylcysteine (NAC) which is an antioxidant inhibited the cell proliferation, migration, and invasion abilities mediated by FOXC1 overexpression, whereas high levels of ROS induced by L-Buthionine-sulfoximine (BSO) rescued the suppression results mediated by FOXC1 knockdown. Our study demonstrated that the overexpression of FOXC1 that was induced by the ROS dependent on the extracellular regulated protein kinases 1 and 2 (ERK1/2)- phospho-ETS Transcription Factor 1 (p-ELK1) pathway. In human HCC tissues, FOXC1 expression was positively correlated with oxidative damage marker 8-hydroxy-2′-deoxyguanosine (8-OHdG), p-ELK1 and DNMT3B expression, but negatively correlated with CTH expression. HCC patients with positive co-expression of 8-OHdG/FOXC1 or p-ELK1/FOXC1 or FOXC1/DNMT3B had the worst prognosis, whereas HCC patients who had positive FOXC1 and negative CTH expression exhibited the worst prognosis.

**Conclusion:**

In a word, we clarify that the positive feedback loop of ROS-FOXC1-cysteine metabolism-ROS is important for promoting liver cancer proliferation and metastasis, and this pathway may provide a prospective clinical treatment approach for HCC.

**Supplementary Information:**

The online version contains supplementary material available at 10.1186/s13046-021-01829-6.

## Background

Metabolic reprogramming is one of the essential features of tumors [1]. Specific metabolic processes can be directly involved in the transformation process or biological processes that support tumor growth [2]. Amino acids play a number of roles in tumor cell growth and survival, including providing carbons to the tricarboxylic acid cycle (TCA cycle), nitrogen to nucleobase synthesis, in maintaining redox homeostasis and other metabolic activities [3]. Meanwhile, amino acids can regulate the development of tumor cells by activating some oncogenes [4]. The liver is the key organ for coordinating metabolic activities, including nitrogen metabolism, detoxification and energy metabolism. In physiological and pathological conditions, the liver provides the energy necessary to maintain the function of different organs. Abnormalities in circulating amino acid metabolite levels were observed in hepatocellular carcinoma (HCC). Recently, clinical studies showed that circulating levels of some biogenic amines and branched-chain, aromatic and glucogenic amino acids were closely associated with the risk of HCC [5]. Therefore, further studies of molecular mechanism underlying amino acid metabolism with HCC which are able to assist with developing novel therapeutic strategies are urgently needed.

Moreover, another risk factor associated with tumorigenesis and tumor progression is an increase in reactive oxygen species (ROS) abundance, which is caused by the production and elimination of an imbalance in the composition of reactive oxygen species [6]. An increase in ROS has been detected in various cancers and has been shown to have multiple roles in activating pro-tumorigenesis signals, driving DNA damage and genetic instability, and enhancing cell survival and proliferation. Counterintuitively ROS can also promote anti-tumorigenic signaling, and trigger tumor cell death induced by oxidative stress. Furthermore, as the important messenger, ROS is associated with the expression of lots of transcription factors [7].

The forkhead box (FOX) protein family consists of a group of evolutionarily- conserved transcription factors characterized by a common DNA-binding domain known as the forkhead box domain [8]. FOX family proteins involve in cell growth, differentiation and other biological processes [9]. The deregulation of Fox family transcription factors is important for the development and progression of tumors [8]. Some researchers have shown that abnormal expression of FOX family protein plays a pivotal role in metabolism. The forkhead box O (FOXO) family participates in the regulation of a large number of biological activities from development, cell signal transduction, tumorigenesis to cell metabolism [10]. FOXO1 induced CIC promoter activity, which involved in lipid synthesis and OXOPHOS [11]. The balance of FOXO and FOXM1 transcription factors integrates Adenosine 5′-monophosphate (AMP)-activated protein kinase (AMPK)-mediated metabolic status and cell cycle regulation through competitive regulation of target genes (including Insulin-like growth factor-1 (IGF1)) in neonatal cardiomyocytes [12]. FOXK1 and FOXK2 are important in the regulation of mitochondrial function, metabolism and apoptosis [13]. FOXO family facilitated the cellular antioxidant defense, and on the other hand, ROS may regulate FOXO activity by regulating phosphorylation, lysine acetylation and ubiquitination and regulate FOXO expression by transcriptional regulation, posttranscriptional regulation and transcriptional coregulators [14]. Therefore, FOX family proteins involve in the development of diseases by altering the activities of cell metabolism and the accumulation of ROS.

As a member of FOX family proteins, FOXC1 was first found to be associated with the ocular dominant genetic disease Axenfeld-Rieger syndrome (ARS) [15]. FOXC1 regulated normal embryonic development and is involved in the development and function of multiple organs [16]. Some research has shown that FOXC1 is positively correlation with poor prognosis of a variety of tumors, including pancreatic ductal adenocarcinoma, acute myeloid leukemia, basal-like breast cancer, gastric cancer and colon cancer [17–21]. Our previous research indicated that FOXC1 is important for promoting HCC metastasis [22, 23]. Nevertheless, whether FOXC1 promotes HCC progression through amino acid metabolism is unclear. Using amino acid metabolism RT^2^ Profiler PCR array (Supplementary Table S1), we found that FOXC1 downregulated cystathionine γ-lyase (CTH) expression, which is associated with cysteine metabolism. Cysteine metabolism is involved in redox balance by regulating the ROS level.

In this study, we demonstrated that FOXC1 upregulated DNA methylases 3B (DNMT3B) to induce DNA hypermethylation of *CTH* promoter and *CTH* gene silencing, which resulted in the decrease of cysteine levels and increases of ROS levels.

Moreover, the high level of ROS increased the expression of FOXC1 through extracellular regulated protein kinases 1 and 2 (ERK1/2)- phospho-ETS Transcription Factor 1 (p-ELK1) pathway, which formed a ROS-FOXC1-cysteine metabolism-ROS positive feedback loop to promote HCC proliferation and metastasis.

## Materials and methods

### Patients and follow-up

This study was approved by the Ethics Committee of Tongji Medical College. All patients provided full consent for the study. Cohort I included 280 adult patients with HCC who underwent curative resection between 2003 and 2005 at the Tongji Hospital of Tongji Medical College (Wuhan, China). Cohort II included 210 adult patients with HCC who underwent curative resection between 2006 and 2008 at the Tongji Hospital of Tongji Medical College (Wuhan, China). A preoperative clinical diagnosis of HCC was based on the diagnostic criteria of the American Association for the Study of Liver Diseases. The inclusion criteria were as follows: (a) distinctive pathologic diagnosis; (b) no preoperative anticancer treatment or distant metastases; (c) curative liver resection; and (d) complete clinicopathologic and follow-up data. The differentiation statuses were graded according to the method of Edmondson and Steine. The pTNM classification for HCC was based on The American Joint Committee on Cancer/International Union Against Cancer staging system (6th edition, 2002). Follow-up data were summarized at the end of December 2013 (Cohort I) and December 2016 (Cohort II, range 4–96 months) respectively. The patients were evaluated every 2–3 months during the first 2 years and every 3–6 months thereafter. All follow-up examinations were performed by physicians who were blinded to the study. During each check-up, the patients were monitored for tumor recurrence by measuring the serum AFP levels and by performing abdominal ultrasound examinations. A computed tomography and/or magnetic resonance imaging examination was performed every 3–6 months, together with a chest radiographic examination. The diagnostic criteria for HCC recurrence were the same as the preoperative criteria. The time to recurrence and overall survival were the primary endpoints. The time to recurrence was calculated from the date of resection to the date of a diagnosis with tumor recurrence. The overall survival was calculated from the date of resection to the date of death or of the last follow-up.

### In vivo metastatic model and bioluminescent imaging

BALB/C nude mice (5 weeks old) were housed under standard conditions and cared for according to the institutional guidelines for animal care. All animal experiments were approved by the Committee on the Use of Live Animals in Teaching and Research (CULATR), Huazhong University of Science and Technology. For in vivo metastasis assay, human luciferase labeled HCC cells (4.0 × 10^6^) in the 100 μl of phosphate-buffered saline that were mixed with 100 μl Matrigel were injected into the right lobes of livers of the nude mice under anesthesia (10 for each group). The in vivo tumor formation and metastases were monitored using the bioluminescence. For in vivo signal detection, D-luciferin (Perkin-Elmer) at 100 mg/kg was injected intraperitonially into the nude mice. Bioluminescent images were captured using an IVIS 100 Imaging System (Xenogeny). At the 9 weeks, the mice were sacrificed and the livers and lungs were collected and underwent histological examination.

### Plasmid construction

Plasmid construction was performed according to standard procedures as outlined in our previous study. The primers are presented in Supplementary Table S7. For instance, the *DNMT3B* promoter construct, (− 1303/+ 109) *DNMT3B*, was generated from human genomic DNA. This construct corresponds to the sequence from − 1303 to + 109 (relative to the transcriptional start site) of the 5′-flanking regions of the human *DNMT3B* gene. It was generated with forward and reverse primers incorporating *KpnI* and *HindIII* sites at the 5′ and 3′-ends, respectively. The polymerase chain reaction (PCR) product was cloned into the *KpnI* and *HindIII* sites of the pGL3-Basic vector (Promega). The 5′-flanking deletion constructs of the *DNMT3B* promoter, (− 924/+ 109) *DNMT3B*, (− 808/+ 109) *DNMT3B*, (− 195/+ 109) *DNMT3B*, were similarly generated using the (− 1303/+ 109) *DNMT3B* construct as the template. The FOXC1 binding sites in the *DNMT3B* promoter were mutated using the QuikChange II Site-Directed Mutagenesis Kit (Stratagene). The constructs were confirmed by DNA sequencing. Other promoter constructs were cloned in the same manner.

### DNA methylation analyses

Genomic DNA was isolated from cells using Genomic DNA Purification kit following the manufacturer’s instructions (Promega). Bisulfite modification of genomic DNA was carried out using the EZDNA methylation Kit (Zymo Research). Briefly, 1 μg of genomic DNA was denatured by NaOH (final concentration, 0.2 mol/L) for 10 min at 37 °C. Hydroquinone (10 mmol/L, 30 μl) and 520 μl of 3 mol/L sodium hydroxide (pH 5) were added, and samples were incubated at 50 °C for 16 h. Modified DNA was purified using Wizard DNA Clean-Up System following the manufacturer’s instructions (Promega) and eluted into 50 μl water. DNA was treated with NaOH (final concentration, 0.3 mol/L) for 5 min at room temperature, ethanol precipitated, and resuspended in 20 μl water. Modified DNA was used immediately or stored at − 20 °C. Primer sequences specific to unmethylated and methylated promoter sequences are listed in Table S6. Each methylation-specific PCR reaction incorporated 100 ng of bisulfite-treated DNA as template, 10 pmol/L of each primer, 100 pmol/L deoxynucleoside triphosphate, 10 PCR buffer, and 1 unit of JumpStart Red Taq Polymerase (Sigma-Aldrich, St. Louis, MO) in a final reaction volume of 25 μl. Cycle conditions were as follows: 95 °C 5 min; 35 cycles (95 °C 30 s, 60 °C 30 s, and 72 °C 30 s); and 72 °C 5 min. Methylation-specific PCR products were analyzed with nondenaturing 6% polyacrylamide gel electrophoresis and stained with ethidium bromide.

### Bisulfite genomic sequencing (BGS)

For bisulfite cloning and sequencing, the amplification was performed using primers designed by the online program (http://www.urogene.org/cgi-bin/methprimer/methprimer.cgi). Bisulfite genomic sequencing was performed to characterize the methylation density in the promoter of CTH using the BigDye Terminator Cycle Sequencing kit version 1.0 (Applied Biosystems). Thirteen CpG sites spanning − 260 to − 20 of the *CTH* gene were evaluated. Sequences were analyzed by using SeqScape software (Applied Biosystems) and Bioedit (http://www.mbio.ncsu.edu/BioEdit/bioedit.html). The nucleotide sequences of the primers used for BGS are provided in Supplementary Table S7.

### ROS levels measurement

The 2′,7′- dichlorofluorescein diacetate (DCFH-DA) (Beyotime, China) was used to detect the ROS levels. Firstly, added DCFH-DA to DMEM (without FBS) in a ratio of 1:1000, and then a volume of 2 mL mixed solution was added to the HCC cells, which were washed 2 times by PBS. Then we put the cells into incubator for 30 min at 37 °C in dark. Then HCC cells were washed again with DMEM (without FBS) for three times, we measured the ROS levels by fluorescence microscope and flow cytometry.

### GSH/GSSG, GSH and cysteine levels measurement

The GSH/GSSG ratio, GSH levels and cysteine levels were detected by Reduced glutathione (GSH) assay kit (Nanjing jiancheng, China), Total glutathione / Oxidized glutathione assay kit (Nanjing jiancheng, China) and Cysteine content test kit (Nanjing jiancheng, China) on the basis of the manufacturer’s instructions. The transfected cells were lysed in culture dishes containing a lysis buffer, and 0.5 ml supernatant was taken from the resulting lysates which were centrifuged. Two ml of the application solution was added and mixed evenly, and then centrifuged at 3000 g for 10 min. Lastly 1 ml of the supernatant was taken for color reaction. The measurements were conducted with a UV–visible spectrophotometer.

A detailed description of the materials and methods used in this study can be found in the online supplementary material.

## Results

### Overexpression of FOXC1 downregulates CTH expression and increases ROS levels

**Fig. 1:**
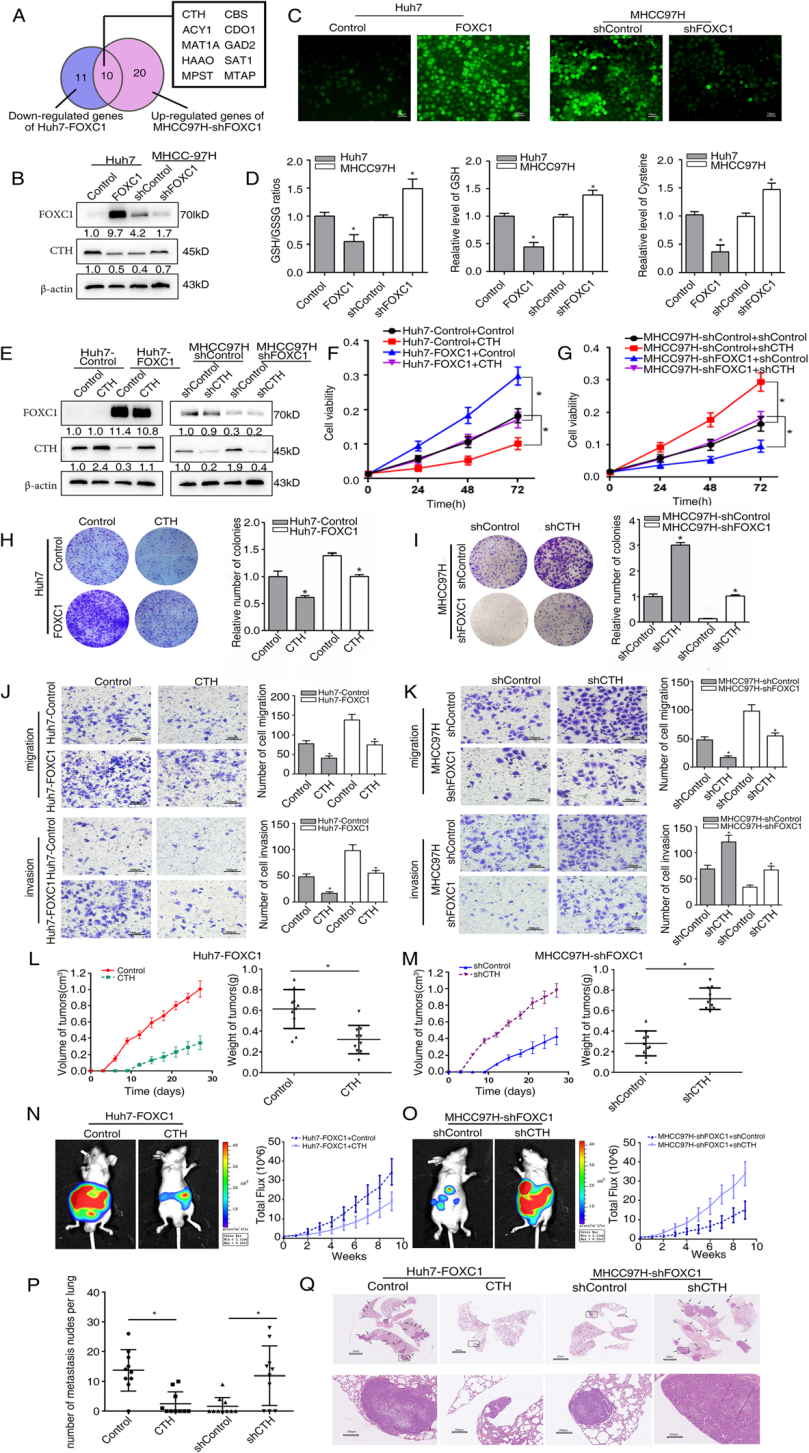
FOXC1 regulates ROS levels and cysteine metabolism through inhibiting CTH expression in HCC cells. **a** Venn diagrams showing the overlap between down-regulated genes in Huh7-FOXC1 and up-regulated genes in MHCC97H-shFOXC1 cells (fold change > 2.0). **b** Western blot analysis showing FOXC1 and CTH protein levels in the HCC cells with upregulation or downregulation of FOXC1. **c** The level of ROS was detected in the indicated HCC cells by immunofluorescence. **d** The GSH/GSSG ratio (left panel), GSH (middle panel) and cysteine (right panel) levels in the indicated cells. **e** Western blot analysis confirmed the stable HCC cell lines have been established. **f**-**g** CCK8 assays detected the proliferation capacities of the indicated HCC cells. **h**-**i** Plate clone formation assay showed the proliferation abilities of the indicated HCC cells. **j**-**k** The migration and invasion abilities of the indicated HCC cells were analyzed by Transwell assays. **l**-**m** In vivo tumorigenesis assays, nude mice were divided into 4 groups (*n* = 10 mice per group). The volume and weight of tumors from different groups were showed. **n**-**o** Representative bioluminescent images and bioluminescence time course of the indicated groups are shown. **p** The number of metastatic lung nodules from different groups. **q** Typical images of hematoxylin and eosin **h** & **e**) stained tissues for each group. Data are represented as mean ± S.D. of three experiments. *P < 0.05

To explore whether FOXC1 participates in the regulation of amino acid metabolism in HCC cells, we first constructed four stable cell lines, Huh7-FOXC1 and MHCC97H-shFOXC1 and their control groups. Then, we performed an amino acid metabolism RT2 Profiler PCR array to examine transcriptome changes mediated by FOXC1 overexpression in Huh7 cells (Supplementary Table S1) and FOXC1 down-expression in MHCC97H cells (Supplementary Table S2). Using twofold as a cut-off to designate differentially expressed genes, 21 out of the 158 amino acid metabolism genes were down-regulated in Huh7 cells which overexpressed FOXC1, while 30 genes were up-regulated in MHCC97H cells which down-expressed FOXC1. Among the overlap of down-regulated genes in Huh7-FOXC1 compared with Huh7-Control cells and up-regulated genes in MHCC97H-shFOXC1 compared with MHCC97H-shControl cells (Fig. 1a), there are 10 genes listed. CTH attracted our attention, which is strongly inhibited by FOXC1 overexpression. We used the western blot analysis to confirm the relationship between CTH and FOXC1, the results demonstrated that up-regulation of FOXC1 expression meaningfully inhibited the expression of CTH, whereas down-regulation of FOXC1 increased the CTH levels (Fig. 1b and Supplementary Fig. S2B). According to the PCR array result, FOXC1 could regulate the mRNA level of cystathionine-β-synthase (CBS), which converted homocysteine to cystathionine [24]. But we found the protein level of CBS have no significant changes with the different expression of FOXC1 by western blot analysis (Supplementary Fig. S2C). Moreover, homocysteine is a part of methionine cycle, which contain methionine, S-adenosylmethionine (SAM) and S-adenosylhomocysteine (SAH). SAM donates its methyl group to DNA, RNA, proteins, or other cellular metabolites, generating SAH [25]. The results of LC/MS showed that SAM levels slightly increased with the upregulation of FOXC1 and decreased by downregulation of FOXC1 (Supplementary Fig. S2D). CTH is the key enzyme for cysteine synthesis (Supplementary Fig. S2A), acted on cystathionine to generate cysteine, and cysteine is the precursor of ROS scavenger [26]. We found that the levels of ROS increased in HCC cells with FOXC1 overexpression and decreased when FOXC1 is down-regulated (Fig. 1c and Supplementary Fig. S2E). Moreover, when FOXC1 overexpressed, the ratio of glutathione (GSH)/ Glutathione (Oxidized) (GSSG) and the level of cysteine and GSH decreased. Correspondingly, the ratio of GSH/GSSG and the level of cysteine and GSH increased when FOXC1 is down-regulated (Fig. 1d).

According to the data from The Cancer Genome Atlas dataset (TCGA), we found that compared with mRNA level of the CTH in normal liver tissues, the mRNA levels of CTH was markedly lessened in HCC specimens. Furthermore, based on TCGA data, the mRNA expression of CTH decreased in cholangiocarcinoma (CHOL), kidney renal clear cell carcinoma (KIRC) and thyroid carcinoma (THCA) tissues (Supplementary Fig. S1A). Human Protein Atlas program database showed high or medium CTH staining intensity in normal liver samples, whereas liver cancer samples showed low staining of CTH by immunohistochemistry (IHC) tissue microarray data (Supplementary Fig. S1B). Kaplan-Meier analysis based on TCGA data showed that HCC patients who had low CTH mRNA level, their overall survival time of were significantly shorter than that with high CTH mRNA level (Supplementary Fig. S1C). These studies suggested that low levels of CTH indicated poor prognosis, and CTH may act as an important tumor suppressor gene (TSG) in human HCC.

### FOXC1 facilitates HCC proliferation and metastasis by inhibiting CTH expression

We upregulated the expression of CTH in Huh7-FOXC1 cells and knocked down CTH in MHCC97H-shFOXC1 cells with lentivirus transfection (Fig. 1e and Supplementary Fig. S2F). The results of CCK8 assays (Fig. 1f), plate clone formation assays (Fig. 1h) and soft agar clone formation assay (Supplementary Fig. S2G) showed that up-regulation of CTH abolished FOXC1-facilitated cell proliferation. Cell cycle analysis showed that overexpressed CTH decreased the cell numbers at S phase which increased by FOXC1 (Supplementary Fig. S2H, upper panel). Meanwhile, CTH attenuated the FOXC1-mediated cell migration and invasion (Fig. 1j). Conversely, knockdown of CTH recovered the proliferative ability inhibited by overexpression of FOXC1 (Fig. 1g and i), increased the cell numbers at the S phase (Supplementary Fig. S2H, lower panel), and reversed the migration and invasion capacities (Fig. 1k).

Up-regulation of CTH significantly inhibited tumor growth induced by Huh7-FOXC1 cells, whereas down-regulated CTH rescued the decreased tumor growth mediated by FOXC1 knockdown by In vivo tumorigenicity assays (Fig. 1l-m). The tumors were detected by Ki67 staining, it also indicated that over-expressed CTH inhibited the proliferation of HCC, and knockdown of CTH had an opposite result (Supplementary Fig. S2I). Consistently, in vivo metastasis assay indicated that overexpression of CTH reduced the HCC metastasis with Huh7-FOXC1 cells (Fig. 1n, p-q, Supplementary Fig. S2J), which prolonged overall survival time (Supplementary Fig. S2K). In contrast, the down-regulation of CTH recused the inhibition of HCC metastasis in the MHCC97H-shFOXC1 group (Fig. 1o-q, Supplementary Fig. S2J). These studies showed that FOXC1 facilitated HCC proliferation and metastasis via inhibiting CTH expression.

### FOXC1 upregulates DNMT3B expression, which results in the DNA hypermethylation of *CTH* promoter and *CTH* gene silencing

**Fig. 2:**
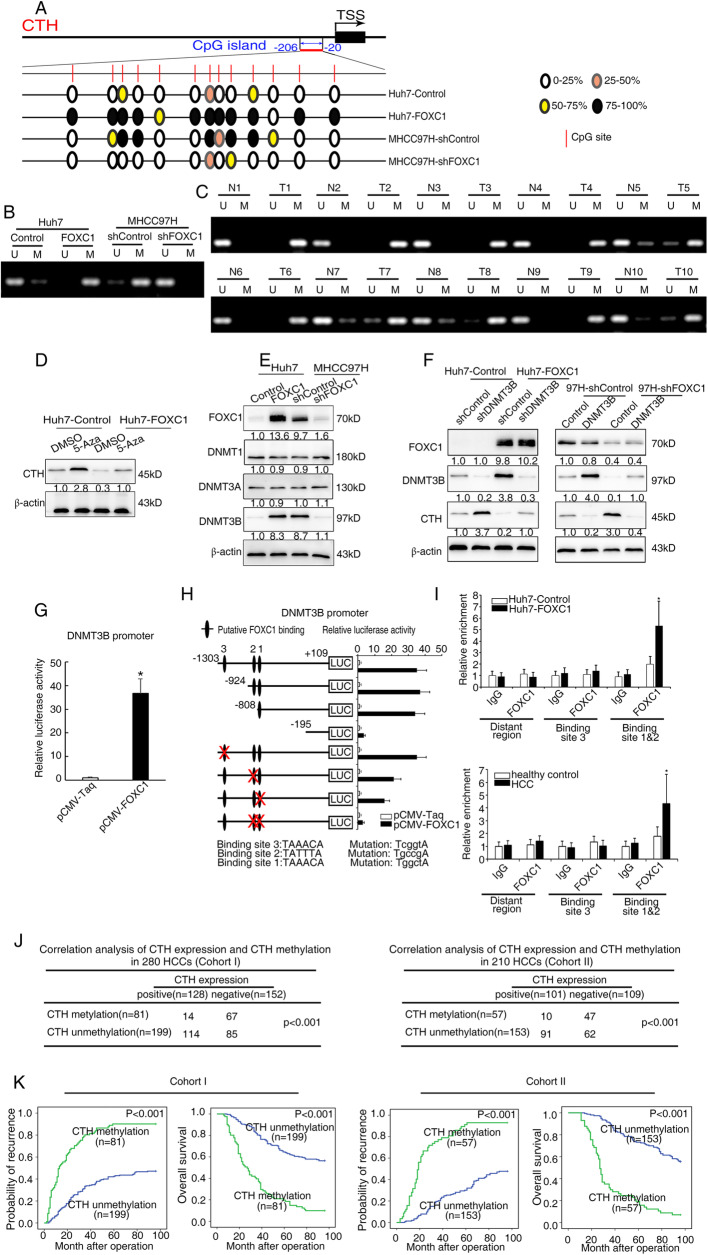
FOXC1 induces DNA hypermethylation of *CTH* promoter and *CTH* gene silencing through upregulating DNMT3B expression. **a** BGS detected the methylation status of the *CTH* promoter in the indicated HCC cells. A typical CpG island is presented at the promoter region of *CTH*. Each red vertical bar represents a single CpG site. The transcription starts site (TSS) is indicated by a curved arrow. Black spots represent 75–100% methylated CpGs, yellow spots denote 50–75% methylated CpGs, orange spots denote 25–50% methylated CpGs and hollow dots represent 0–25% methylated CpGs. **b**-**c** DNA methylation status of the *CTH* promoter was examined by MSP in the indicated HCC cells and in 10 pairs of HCC tissues (T1-T10) and tumor-adjacent tissues (N1-N10). U, unmethylated DNA; M, methylated DNA. **d** After the cells were treated with 5-Aza (5 μM, 72 h), western blots were used to detect the protein levels of CTH. **e**-**f** Western blotting analysis of DNMT1, DNMT3A, DNMT3B and FOXC1 expression in the indicated cells. **g** Relative luciferase activity in Huh7 cells after the co-transfection of a construct containing the *DNMT3B* promoter with an FOXC1-overexpressing construct was examined by luciferase reporter assay. **h** *DNMT3B* promoter constructs with serially truncated and mutated sequences which were cloned into pGL3-luciferase reporter plasmids were transfected into Huh7 cells. Then, pCMV-FOXC1 plasmids were co-transfected to determine the relative activity of luciferase. The structure of the diagram is shown (left) and the histogram shows the relative level of luciferase activity in each sample (right). **i** ChIP assays clarified the direct binding of FOXC1 to the *DNMT3B* promoter in Huh7-FOXC1 cells (upper panel) and the enriched binding of endogenous FOXC1 to the *DNMT3B* promoter in primary HCC tissues (lower panel). Hepatocytes were isolated from the liver tissues of HCC patients (*n* = 6) and healthy controls (*n* = 3). **j** Correlation analysis of the expression of CTH and promoter methylation status of *CTH* in HCC patients in cohort I (left) and cohort II (right). **k** Kaplan-Meier analysis of the correlation between promoter methylation status of CTH and the recurrence or overall survival in patients with HCC in cohort I (left) and cohort II (right). Data are represented as mean ± S.D. of three experiments. *P < 0.05

Epigenetic modifications such as DNA methylation of TSG promoters contribute to the progression and metastasis of HCC [27]. Recent studies indicated that the inhibition of *CTH* gene transcription resulted from DNA hypermethylation of CpG rich region in the *CTH* promoter [28–30]. To determine whether promoter DNA hypermethylation results in *CTH* gene silencing in FOXC-overexpressing HCC cells, the methylation status of CpG island in the *CTH* promoter was analyzed by bisulfite genome sequencing (BGS) analysis, which covered 13 CpG sites from − 206 to − 20 of the *CTH* promoter (Fig. 2a). Hypermethylation at CpG sites in the *CTH* promoter region was detected in Huh7-FOXC1 cells, whereas less methylation was detected in MHCC97H-shFOXC1 (Fig. 2a and b). Compared with adjacent nontumor tissues, HCC tissues showed much higher methylation levels at CpG sites in the *CTH* promoter region (Fig. 2c). In addition, treatment with a demethylation agent, the DNA methyltransferase inhibitor 5-aza-2′-deoxycytidine (5-Aza), restored CTH expression in Huh7-FOXC1 cells, indicating that promoter DNA hypermethylation contributes to the transcriptional silencing of *CTH* gene (Fig. 2d and Supplementary Fig. S3A).

Besides DNA methylation, the metabolism-related genes could be regulated by histone acetylation [31]. We assumed that CTH may also regulated by histone acetylation. To verify the assumption, we use Trichostatin A (TSA), the inhibitor of histone deacetylase, to detect the influence of histone acetylation on expression of CTH. We found that TSA treatment can increase the expression of CTH in Huh7-Control cells, but have no significant changes in Huh7-FOXC1 cells (Supplementary Fig. S3B). It indicated that histone acetylation didn’t contribute to the expression of CTH mediated by FOXC1.

As the important DNA methyltransferases, DNMT3A and DNMT3B are involved in de novo methylation patterns, which are maintained by DNMT1 [32]. Therefore, the DNA hypermethylation of *CTH* promoter may be induced by DNMTs. We found that the expression of DNMT3B is increased in Huh7-FOXC1 cells, whereas DNMT3B expression is downregulated in MHCC97H-shFOXC1 cells. However, the expression of DNMT1 and DNMT3A had no significant change in Huh7-FOXC1 and MHCC97H-shFOXC1 cells compared to control cells (Fig. 2e and Supplementary Fig. S3C). Moreover, ectopically overexpression of DNMT3B decreased the expression of CTH in MHCC97H-shFOXC1 cells, whereas knockdown of DNMT3B increased CTH expression in Huh7-FOXC1 cells (Fig. 2f and Supplementary Fig. S3D). Furthermore, the BSP results showed that knockdown DNMT3B expression could decrease the DNA methylation levels of CTH mediated by upregulation of FOXC1, whereas overexpression of DNMT3B had the opposite results (Supplementary Fig. S3E). These studies suggested that FOXC1 upregulated DNMT3B expression, which resulted in *CTH* gene silencing in HCC cells.

### DNMT3B is a direct transcriptional target of FOXC1

The mechanism by which FOXC1 upregulated DNMT3B expression is still unclear. We hypothesized that FOXC1 may directly transactivate *DNMT3B*. A dual-luciferase reporter assay showed that overexpression of FOXC1 enhanced the transcription of the luciferase reporter in the *DNMT3B* plasmid constructs compared to that of the controls (Fig. 2g). In order to clarify the regulation mechanism of DNMT3B, the promoter sequence of *DNMT3B* was analyzed and we found 3 putative *FOXC1* binding motifs. To further confirm whether FOXC1 targets these potential binding sites to regulate DNMT3B, we designed a series of reporter plasmids containing truncated or mutated DNMT3B promoter sequences and used them in luciferase reporter assay. Huh7 cells were transfected with these plasmids to assess their reaction to FOXC1 overexpression. The results suggested that FOXC1-induced luciferase reporter expression was significantly eliminated by deletion in the − 195 ~ + 109 bp region compared to that of the controls. Consistently, compared with the control group, mutations at the putative binding sites 2&1 of the *DNMT3B* promoter significantly reduced the activity of the FOXC1 overexpressed luciferase reporter gene, suggesting that these binding sites were essential for FOXC1 transactivation (Fig. 2h). A chromatin immunoprecipitation (ChIP) assay demonstrated the direct binding of FOXC1 to the putative site in the *DNMT3B* promoter in Huh7-FOXC1 cells (Fig. 2i upper). Moreover, we investigated whether FOXC1 binds to the same regions in patient samples, and the results revealed that compared to healthy controls, FOXC1 binding sites in the HCC samples were indeed enriched in these regions. (Fig. 2i, lower).

Then we examined the protein levels of CTH and promoter methylation levels of *CTH* in two independent cohorts of human HCC tissues. Microvascular invasion, poor tumor differentiation, and higher TNM stage were positively associated with the depletion of CTH (Table 1). Loss of CTH expression was an independent and meaningful risk factor for recurrence and reduced survival according to the multivariate analysis (Table 2). The expression of CTH was inversely correlated with promoter methylation levels of CTH in both cohorts (Fig. 2j). In addition, promoter methylation of *CTH* gene was positively correlated with aggressive tumor behaviors (Supplementary Table S3). HCC patients with promoter methylation of *CTH* had higher recurrence rates and shorter overall survival time than HCC patients without promoter methylation of *CTH* (Fig. 2k).

**Table 1:** Correlation between CTH expression and clinicopathological characteristics of HCCs in two independent cohorts of human HCC tissues

Clinicopathological variables	Cohort I		*P* Value	Cohort II		*P* Value
	Tumor CTH expression			Tumor CTH expression		
	Negative (*n* = 152)	Positive (*n* = 128)		Negative (*n* = 109)	Positive (*n* = 101)	
Age	51.63 (9.148)	52.85 (10.966)	0.283	50.95 (10.857)	54.59 (9.943)	0.546
Sex						
female	25	20	0.872	20	19	1.000
male	127	108		89	82	
Serum AFP						
≤ 20 ng/ml	24	25	0.433	19	31	0.034
> 20 ng/ml	128	103		90	70	
Virus infection						
HBV	108	85	0807	76	82	0.129
HCV	22	22		11	8	
HBV + HCV	9	7		5	5	
none	13	14		17	6	
Cirrrhosis						
absent	38	41	0.230	28	29	0.644
present	114	87		81	72	
Child-pugh score						
Class A	122	114	0.049	81	77	0.752
Class B	30	14		28	24	
Tumor number						
single	91	100	0.001	56	68	0.024
multiple	61	28		53	33	
Maximal tumor size						
≤ 5 cm	78	84	0.021	45	54	0.097
> 5 cm	74	44		64	47	
Tumor encapsulation						
absent	54	21	< 0.001	62	24	< 0.001
present	98	107		47	77	
Microvascular invasion						
absent	81	91	0.003	41	75	< 0.001
present	71	37		68	26	
Tumor differentiation						
I-II	100	107	0.001	76	91	< 0.001
III-IV	52	21		33	10	
TNM stage						
I-II	101	122	< 0.001	73	97	< 0.001
III	51	6		36	4

**Table 2:** Univariate and Multivariate Analysis of Factors Associated with Time To Recurrence and Overall Survival in Cohort I HCC Patients (*n* = 280) and Cohort II HCC Patients (*n* = 210)

Cohort I (*n* = 280)	Time To Recurrence		Overall Survival	
Clinical Variables	HR(95%CI)	*P* value	HR(95%CI)	*P* value
**Univariate Analysis**				
Age	0.994 (0.979–1.009)	0.427	0.989 (0.973–1.004)	0.152
Sex (female versus male)	0.861 (0.574–1.293)	0.861	0.902 (0.592–1.373)	0.630
Serum AFP (≤20 versus > 20 ng/ml)	1.418 (0.927–2.170)	0.108	1.301 (0.849–1.994)	0.227
Virus infection (no versus yes)	0.986 (0.796–1.222)	0.896	1.002 (0.808–1.243)	0.986
Cirrhosis (absent versus present)	1.039 (0.741–1.456)	0.825	1.132 (0.797–1.606)	0.489
Child-pugh score (A versus B)	1.254 (0.835–1.884)	0.274	1.247 (0.824–1.887)	0.296
Tumor number (single versus multiple)	2.596 (1.903–3.540)	< 0.001	0.343 (0.250–0.470)	< 0.001
Maximal tumor size (≤5 versus > 5 cm)	0.583 (0.482–0.706)	0.013	0.696 (0.509–0.951)	0.023
Tumor encapsulation (absent versus present)	0.341 (0.248–0.469)	< 0.001	3.065 (2.221–4.230)	< 0.001
Microvascular invasion (absent versus present)	2.338 (1.720–3.179)	< 0.001	0.405 (0.296–0.554)	< 0.001
Tumor differentiation (I-II versus III-IV)	0.295 (0.241–0.361)	< 0.001	0.317 (0.229–0.439)	< 0.001
TNM stage (I-II versus III)	3.032 (2.193–4.191)	< 0.001	0.145 (0.102–0.205)	< 0.001
CTH (negative versus positive)	6.289 (4.444–8.901)	< 0.001	3.733 (2.629–5.299)	< 0.001
**Multivariate analysis**				
Tumor number (single versus multiple)	0.707 (0.473–1.057)	0.091	0.616 (0.413–0.920)	0.018
Maximal tumor size (≤5 versus > 5 cm)	1.050 (0.749–1.471)	0.778	1.085 (0.766–1.537)	0.645
Tumor encapsulation (absent versus present)	1.448 (0.937–2.238)	0.096	1.392 (0.889–2.178)	0.148
Microvascular invasion (absent versus present)	0.674 (0.469–0.968)	0.033	0.631 (0.436–0.912)	0.014
Tumor differentiation (I-II versus III-IV)	0.858 (0.514–1.435)	0.560	0.933 (0.548–1.587)	0.798
TNM stage (I-II versus III)	0.357 (0.197–0.645)	0.001	0.335 (0.184–0.611)	< 0.001
CTH (negative versus positive)	2.759 (1.941–3.922)	< 0.001	2.969 (2.054–4.293)	< 0.001
Cohort II (n = 210)	Time To Recurrence		Overall Survival	
Clinical Variables	HR(95%CI)	P value	HR(95%CI)	P value
**Univariate Analysis**				
Age	0.987 (0.970–1.004)	0.122	0.985 (0.968–1.002)	0.076
Sex (female versus male)	0.769 (0.499–1.184)	0.232	0.722 (0.468–1.115)	0.722
Serum AFP (≤20 versus > 20 ng/ml)	1.199 (0.788–1.825)	0.397	1.248 (0.809–1.927)	0.316
Virus infection (no versus yes)	0.959 (0.700–1.313)	0.792	0.923 (0.669–1.275)	0.628
Cirrhosis (absent versus present)	0.873 (0.595–1.282)	0.489	0.860 (0.583–1.271)	0.450
Child-pugh score (A versus B)	0.973 (0.649–1.460)	0.896	0.981 (0.649–1.482)	0.927
Tumor number (single versus multiple)	1.984 (1.398–2.817)	< 0.001	0.484 (0.338–0.691)	< 0.001
Maximal tumor size (≤5 versus > 5 cm)	1.328 (0.934–1.888)	0.114	0.705 (0.491–1.012)	0.058
Tumor encapsulation (absent versus present)	0.354 (0.249–0.505)	< 0.001	3.085 (2.148–4.431)	< 0.001
Microvascular invasion (absent versus present)	2.319 (1.629–3.301)	< 0.001	0.389 (0.271–0.559)	< 0.001
Tumor differentiation (I-II versus III-IV)	2.128 (1.422–3.184)	< 0.001	0.431 (0.287–0.647)	< 0.001
TNM stage (I-II versus III)	7.507 (4.967–11.345)	< 0.001	0.124 (0.082–0.188)	< 0.001
CTH (negative versus positive)	0.261 (0.178–0.382)	< 0.001	4.409 (2.950–6.588)	< 0.001
**Multivariate analysis**				
Tumor number (single versus multiple)	1.011 (0.667–1.534)	0.958	0.986 (0.638–1.523)	0.949
Maximal tumor size (≤5 versus > 5 cm)	0.856 (0.510–1.436)	0.556	0.787 (0.462–1.340)	0.377
Tumor encapsulation (absent versus present)	1.679 (1.012–2.786)	0.045	1.636 (0.976–2.742)	0.062
Microvascular invasion (absent versus present)	1.111 (0.660–1.871)	0.693	1.000 (0.591–1.694)	1.000
Tumor differentiation (I-II versus III-IV)	0.851 (0.547–1.324)	0.475	0.778 (0.499–1.212)	0.267
TNM stage (I-II versus III)	0.173 (0.094–0.321)	0.001	0.172 (0.091–0.323)	< 0.001
CTH (negative versus positive)	3.403 (2.243–5.164)	< 0.001	3.816 (2.467–5.903)	< 0.001

### DNMT3B is critical for FOXC1-induced HCC proliferation and metastasis

**Fig. 3:**
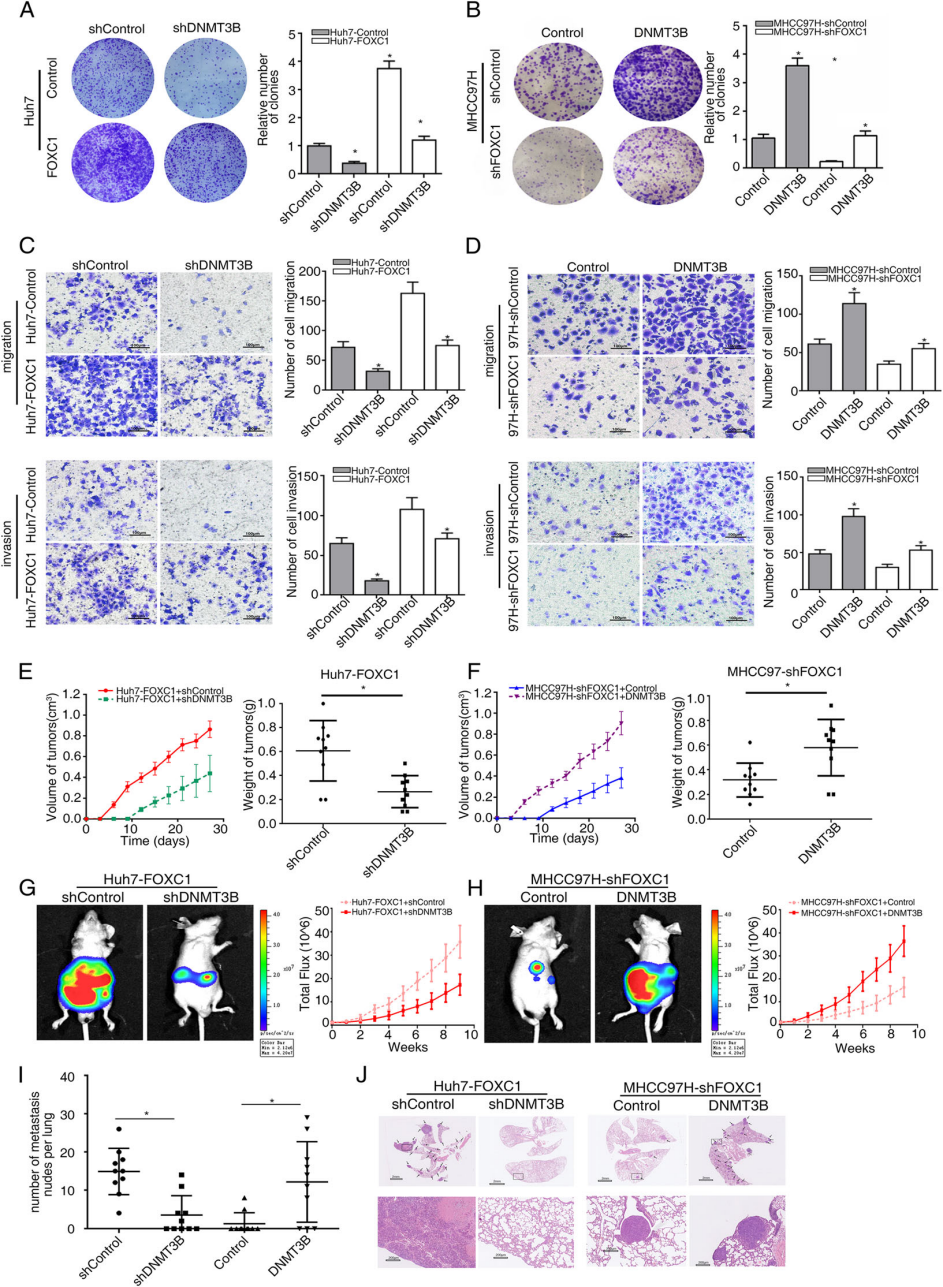
DNMT3B is critical for FOXC1-induced HCC proliferation and metastasis. **a**-**b** Plate clone formation assay showed the proliferation abilities of the indicated HCC cells. **c**-**d** The migration and invasion abilities of the indicated HCC cells were analyzed by Transwell assays. **e**-**f** In vivo tumorigenesis assays, nude mice were divided into 4 groups (*n* = 10 mice per group). The volume and weight of tumors from different groups were showed. **g**-**h** Representative bioluminescent images and bioluminescence time course of the indicated groups are shown. **i** The number of metastatic lung nodules from different groups. **j** Typical images of hematoxylin and eosin (H&E) stained tissues for each group. Data are represented as mean ± S.D. of three experiments. *P < 0.05

To explore whether DNMT3B was involved in FOXC1-mediated HCC proliferation and metastasis, we knocked down DNMT3B in Huh7-FOXC1 cells and ectopically upregulated DNMT3B expression in MHCC97H-shFOXC1 cells with lentivirus transfection (Fig. 2f). Down-regulation of DNMT3B inhibited FOXC1-facilitated HCC proliferation, migration, and invasion abilities, while up-regulation of DNMT3B had the opposite result (Fig. 3a-d and Supplementary Fig. S4A-F). In vivo tumorigenicity assays suggested that down-regulated DNMT3B significantly inhibited tumor growth induced by Huh7-FOXC1 cells (Fig. 3e), whereas upregulation of DNMT3B salvaged the suppression tumor growth induced by FOXC1 knockdown (Fig. 3f). Representative Ki67-stained images are shown (Supplementary Fig. S4G). In vivo metastatic assay suggested that down-regulated DNMT3B eliminated HCC metastasis with Huh7-FOXC1 cells, which prolonged overall survival time. In contrast, overexpression of DNMT3B rescued the inhibition of HCC metastasis in the MHCC97H-shFOXC1 groups (Fig. 3g-i and Supplementary Fig. S4H-I). Representative H&E-stained images are shown (Fig. 3j). These results identified that FOXC1 facilitated HCC proliferation and metastasis through upregulating DNMT3B expression.

### FOXC1 expression is positively correlated with DNMT3B expression and negatively correlated with CTH expression in human HCC tissues

**Fig. 4:**
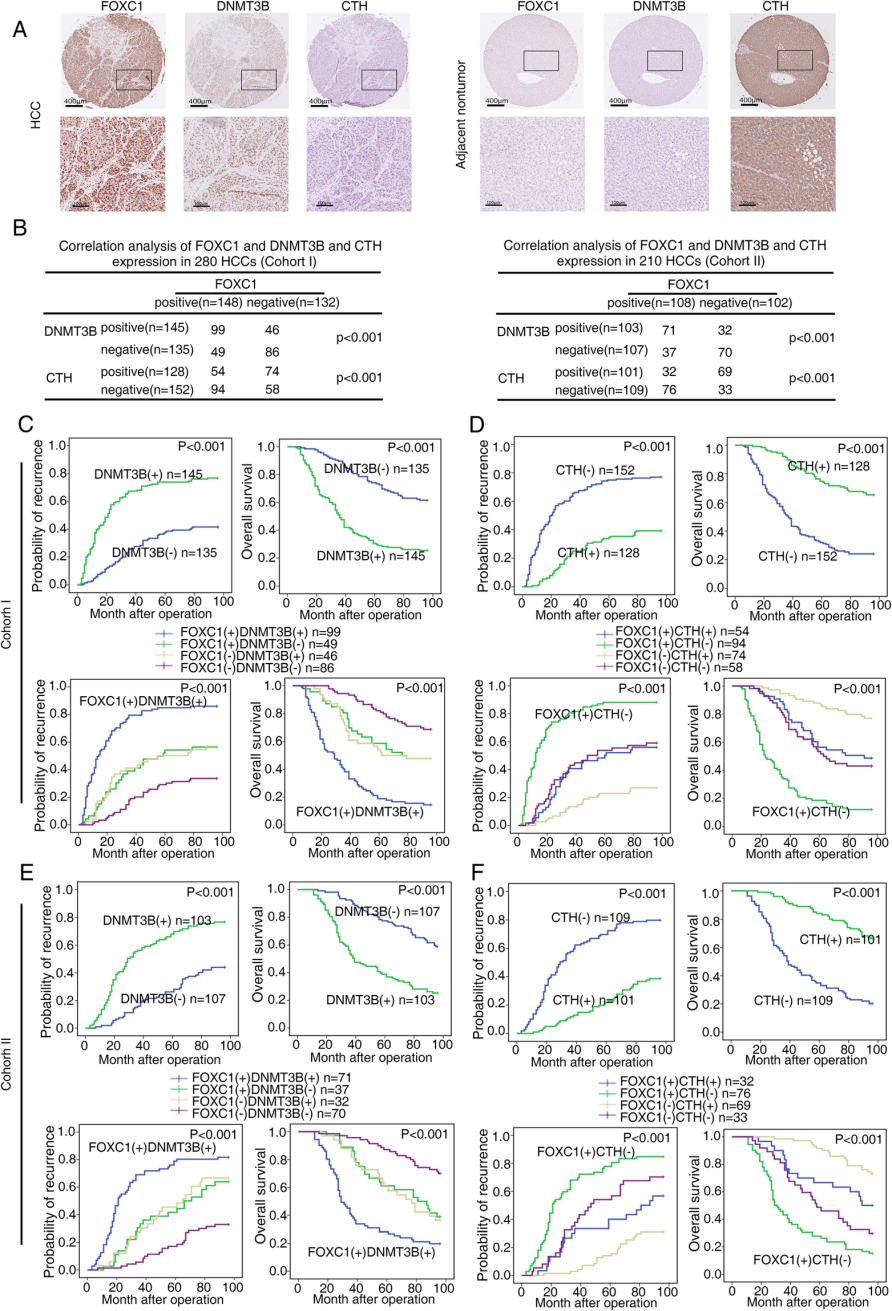
FOXC1 expression is positively correlated with DNMT3B expression and negatively correlated with CTH expression in human HCC tissues. **a** Representative images showing the FOXC1, DNMT3B and CTH expression in adjacent nontumor tissues and HCC tissues evaluated by IHC staining. Scale bars represent 400 μm (low magnification) and 100 μm (high magnification). **b** Correlation analysis of the expression of FOXC1 and DNMT3B or CTH in HCC patients in cohort I (left) and cohort II (right). **c** and **e** Kaplan-Meier analysis of the association between DNMT3B expression or the concurrent FOXC1 and DNMT3B expression and the recurrence rate or overall survival in both cohorts. **d** and **f** Kaplan-Meier analysis of the association between CTH expression or the concurrent FOXC1 and CTH expression and the recurrence rate or overall survival in both cohorts

Immunohistochemical (IHC) analysis was used to verify the clinical relevance of FOXC1 and DNMT3B or CTH in human HCC specimens from two independent cohorts. The representative images of FOXC1, DNMT3B, and CTH expression were showed by IHC staining (Fig. 4a). Compared to adjacent nontumor tissues, DNMT3B expression was observably increased in HCC tissues, whereas CTH expression was observably decreased in HCC tissues. FOXC1 expression was positively associated with DNMT3B expression but negatively associated with CTH expression in both cohorts (Fig. 4b). Up-expression of DNMT3B was positively correlation with poor prognosis (Fig. 4c and e, upper panel) and aggressive tumor behavior (Supplementary Table S4). Both TCGA database and Human Protein Atlas program database showed that compared to the normal liver tissues, the mRNA and protein levels of DNMT3B in liver cancer tissues were much higher (Supplementary Fig. S1D-E). Kaplan–Meier analysis based on TCGA data displayed that compared to HCC patients with low DNMT3B mRNA levels, patients who have high DNMT3B mRNA levels had shorter overall survival time and disease-free survival time (Supplementary Fig. S1F). In addition, the reduced expression of CTH indicated poor prognosis (Fig. 4d and f upper panel). Patients who had both of high expression of FOXC1 and DNMT3B endured the highest recurrence rates and lowest overall survival time (Fig. 4c and e, lower panel). Consistently, patients with the FOXC1(+)/CTH (−) expression pattern had the highest recurrence rates and lowest overall survival time (Fig. 4d and f, lower panel).

### High levels of ROS are critical for FOXC1-mediated HCC cell proliferation, migration and invasion

**Fig. 5:**
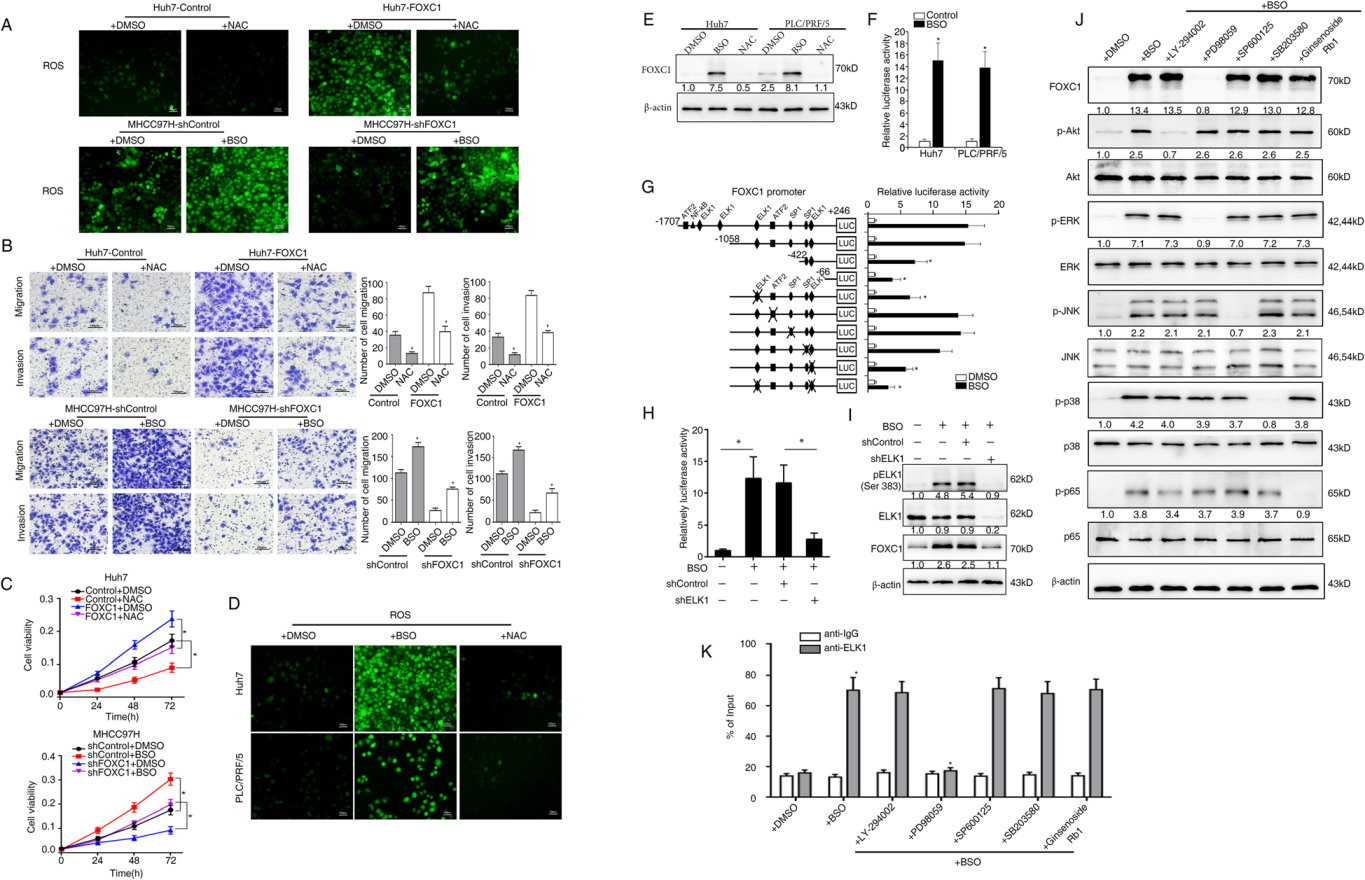
ROS upregulates FOXC1 expression through ERK1/2-p-ELK1 pathway. **a** After the cells were treated with BSO (30 μM, 24 h) or NAC (0.2 mM, 24 h), the ROS levels were detected by immunofluorescence. **b** After the cells were treated with BSO (30 μM, 24 h) or NAC (0.2 mM, 24 h), the migration and invasion abilities of the indicated HCC cells were detected by Transwell assays. **c** CCK8 assays indicated that NAC (upper panel) abolished FOXC1-mediated cell proliferation and BSO (lower panel) rescued cell proliferation inhibition by FOXC1 knockdown. **d** Immunofluorescence detection of the ROS levels when PLC/PRF/5 and Huh7 cells were preprocessed with BSO (30 μM, 24 h) or NAC (0.2 mM, 24 h). **e** After PLC/PRF/5 and Huh7 cells were treated with BSO (30 μM, 24 h) or NAC (0.2 mM, 24 h), western blot was used to detect the protein level of FOXC1. **f** Huh7 cells followed by BSO disposing (30 μM, 24 h) were transfected with *FOXC1* promoter construct, and then to examine the relative luciferase activity. **g** Continuous truncated and mutated *FOXC1* promoter constructs were transfected into cells stimulated by BSO to determine the relative activity of luciferase. **h** ROS induces FOXC1 expression by activating ELK1. Huh7 cells followed by BSO disposing and infected with the lentivirus LV-shELK1 were transinfected with the *FOXC1* promoter construct, and then use luciferase reporter assays to detect the relative luciferase activity. **i** Huh7 cells were treated with BSO and then transfected with lentivirus LV-shELK1 to inhibit the expression of ELK1. After treated with BSO for twenty-four hours, western blotting analysis detected the protein levels of FOXC1 expression. **j** The protein levels of total and phosphorylated Akt, ERK1/2, JNK, p38 and p65 and FOXC1 expression upon BSO stimulation and indicated inhibitor treatment were analyzed by western blot. (K) ChIP assays demonstrated that ROS promoted the direct binding of ELK1 to the *FOXC1* promoter through the ERK1/2 pathway. Data are represented as mean ± S.D. of three experiments. *P < 0.05

To explore whether ROS regulate FOXC1-mediated HCC proliferation, migration and invasion, N-acetylcysteine (NAC) which is an antioxidant [33] and L-Buthionine-sulfoximine (BSO) which decreases GSH levels [34], were used to treat Huh7 -FOXC1 cells and MHCC97H-shFOXC1 cells, respectively. NAC (0.2 mM) treatment abolished high levels of ROS induced by FOXC1 overexpression, whereas BSO (30 μM) treatment rescued the decreased ROS levels induced by FOXC1 knockdown (Fig. 5a and Supplementary Fig. S5A-B). Meanwhile, low levels of ROS induced by NAC inhibited the cell proliferation, migration, and invasion abilities mediated by FOXC1 overexpression and high levels of ROS induced by BSO rescued the suppression results mediated by FOXC1 knockdown (Fig. 5b-c).

### High levels of ROS upregulates FOXC1 expression through ERK1/2-pELK1 pathway

We have elucidated that overexpression of FOXC1 increased ROS levels by inhibiting cysteine metabolism and ROS promotes HCC progression and metastasis. Meanwhile, we previously reported that FOXC1 is over-expressed in human HCC tissues and promoted HCC progression and metastasis [22, 23]. Therefore, we determined whether high levels of ROS regulate FOXC1 expression in HCC cells. Treatment with BSO and NAC could increase and decrease ROS levels respectively (Fig. 5d and Supplementary Fig. S5C). BSO treatment significantly increased FOXC1 expression and NAC treatment decreased FOXC1 expression at protein level in HCC cells (Fig. 5e and Supplementary Fig. S5D). Notably, with the stimulation of BSO, *FOXC1* promoter activity was significantly increased, indicating that high levels ROS transactivated *FOXC1* promoter to elevated the expression of FOXC1 (Fig. 5f).

To verify the accurate location of cis-regulatory elements in the *FOXC1* promoter sequence that reacted to ROS, we generated a series of truncated mutants of the *FOXC1* promoter (Fig. 5g). Dramatic suppression in FOXC1 promoter activity were found in mutants with two deletions from nt-1058 to nt-422 and nt-422 to nt-66, suggesting that these sequences are important in allowing ROS-enhanced FOXC1 activation. The prior region contains one *ELK1* binding site, one specificity protein 1 (*SP1*) binding site and one *ATF2* binding site. And the later region contains one *SP1* binding site and *ELK1* binding site. Notably, site-directed mutagenesis at the *ELK1* binding sites inhibited the ROS-promoted FOXC1 activity, while no effect was found for mutations at the *SP1* binding site and *ATF2* binding site (Fig. 5g). Meanwhile, knockdown of ELK1 abolished *FOXC1* promoter activity which is increased by BSO (Fig. 5h). We found that BSO didn’t increase the expression of ELK1 but activate phosphorylation of ELK1 when activated FOXC1 expression (Fig. 5i and Supplementary Fig. S5E).

ROS activates Nuclear factor kB (NF-kB), c-Jun N-terminal kinase (JNK), ERK1/2, p38 kinases and Phosphoinositide 3-kinase (PI3K)/ protein kinase B (Akt) pathways to promote cancer progression [35]. To verify that ROS regulate FOXC1 expression via which pathway, we treated cells with PI3K, p38 kinases, JNK, NF-κB and ERK1/2 inhibitors. Preconditioning cells with ERK1/2 inhibitor decreased ROS-induced FOXC1 expression (Fig. 5j and Supplementary Fig. S5F). Nevertheless, there was on effect on ROS regulating FOXC1 expression when cells were pretreated with other inhibitors. Further, ChIP assays showed that the ERK1/2 inhibitor dramatically weakened *ELK1* binding to the *FOXC1* promoter, while there were on significant changes on the binding of *ELK1* to the *FOXC1* promoter by other inhibitors (Fig. 5k). These results demonstrated that ROS induced FOXC1 over-expression via the ERK1/2-p-ELK1 signaling pathway.

**Fig. 6:**
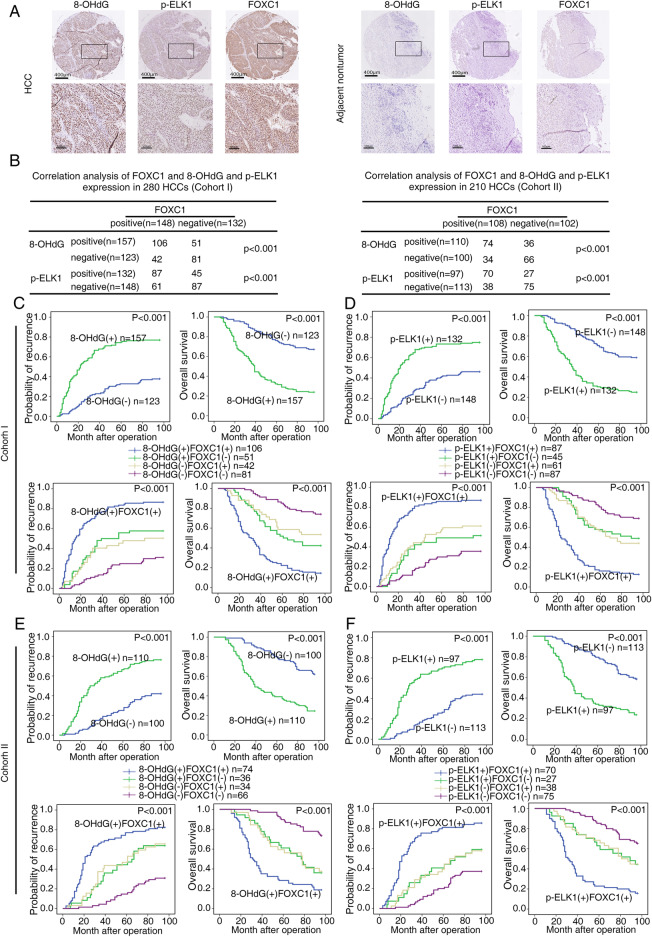
FOXC1 expression is positively correlated with 8-OHdG and p-ELK1 expression in human HCC tissues. **a** Representative images showing the 8-OHdG level, p-ELK1 level and FOXC1 expression in adjacent nontumor tissues and HCC tissues evaluated by IHC staining. Scale bars represent 400 μm (low magnification) and 100 μm (high magnification). **b** Association between the level of 8-OHdG or p-ELK1 and FOXC1 in HCC patients in cohort I (left) and cohort II (right). **c**-**e** Kaplan-Meier analysis of the correlation between the recurrence rate or overall survival time and 8-OHdG level or the concurrent 8-OHdG and FOXC1 expression in both cohorts. **d**-**f** Kaplan-Meier analysis of the correlation between the recurrence rate or overall survival time and p-ELK1 expression or the concurrent p-ELK1 and FOXC1 expression in both cohorts

8-OHdG is the oxidative damage marker [36]. IHC was used to detect 8-OHdG, phospho-ELK1 (activated ELK1) expression in two independent cohorts of human HCC tissue arrays. Compared to adjacent nontumor tissues, both 8-OHdG and p-ELK1 levels was markedly elevated in HCC tissues. 8-OHdG and p-ELK1 levels were localized in the nucleus (Fig. 6a). FOXC1 expression was positively associated with both 8-OHdG level and p-ELK1 expression in two cohorts (Fig. 6b). patients with elevated 8-OHdG formation (Fig. 6c and e, upper panel) and high expression of p-ELK1(Fig. 6d and f, upper panel), compared to patients with low level of 8-OHdG and p-ELK1, had shorter overall survival and higher recurrence rates. Elevated level of both 8-OHdG and p-ELK1 were positively correlated with loss of tumor encapsulation, microvascular invasion, poor tumor differentiation, and a higher TNM stage (Supplementary Table S5, S6). Furthermore, patients with positive co-expression of 8-OHdG (Fig. 6c and e, lower panel) and FOXC1 or co-expression of p-ELK1 ((Fig. 6d and f, upper panel)) and FOXC1 had the highest recurrence rates and lowest overall survival times.

## Discussion

Hepatocellular carcinoma has a high rate of cancer-related death [37]. Early recurrence and metastasis often occur after radical resection of HCC, which leads to poor prognosis of HCC patients [38]. Thus, the molecular mechanism of HCC metastasis needs to be further elucidated to develop novel therapeutic strategies. As we all know, dysregulated metabolism is a hallmark of cancer, manifested through alterations in metabolites [1]. Studies reported that the progression of HCC is associated with the aberrant levels of amino acids [5]. However, how deregulation of amino acid metabolism affect HCC proliferation and metastasis remains unclear. Cysteine is a semi-essential amino acid, which can be acquired from the diet or synthesized from methionine through the reverse transsulfuration pathway by cystathionine γ-lyase (CTH) [26]. Depletion of CTH results in oxidative stress, vascular defects, abnormal stress responses, and hyperhomocysteinemia [39, 40]. Cysteine produced the ROS scavenger glutathione, which decrease ROS levels, by the enzymes GCLC, GCLM [41]. Cysteine levels determines the GSH levels and influence the ROS levels. Uncontrolled increase in ROS production result in damage to large molecules such as DNA, proteins and lipids, leading to genomic instability and changes in cell growth [42]. As a messenger, ROS also affect several transcription factors, such as HIF1-α, NF-κB, AP-1, NRF2, which are important for cancer development [6].

FOX family play different roles in HCC development and progression. FOXA1 and FOXA2 have controversial roles in HCC, they can act as a target of no-coding RNAs to promote HCC [43, 44] and suppressing PIK3R1 to inhibit the HCC proliferation, migration and invasion [45]. FOXC transcription factors promote the progression of HCC by regulating MMPs [23, 46]. FOXM1 could regulate the cell cycle and EMT related molecules expression to facilitate HCC progression [47]. FOXO1 and FOXO3a act as both of suppressor and oncogene in HCC determined by the phosphorylation status and subcellular location [48, 49]. FOXP3 could inhibit or promote HCC by regulating the tumor microenvironment, mutation, post-translational modification or alternative RNA splicing [50–53]. Our previous studies showed that FOXC1 is important for promoting HCC metastasis [22, 23]. In this study, our amino acid metabolism RT^2^ Profiler PCR array indicated that FOXC1 inhibited CTH expression, which is involved in cysteine pathways. Overexpression of FOXC1 decreased the cysteine level and increased the ROS level in HCC cells. Overexpression of CTH significantly decreased FOXC1-mediated HCC proliferation and metastasis, while knockdown of CTH recused the suppression of cell proliferation and metastasis that was induced by the down-regulation of FOXC1. In human HCC tissues, FOXC1 expression was negatively associated with CTH expression, and the patients with high expression of FOXC1 and low expression of CTH exhibited the worst prognosis. These results indicated that FOXC1 facilitated HCC proliferation and metastasis through inhibiting CTH expression and increasing ROS levels.

Although we found that overexpression of FOXC1 inhibited CTH expression, its underlying mechanism remains unclear. Some researchers reported that the attenuation of *CTH* gene transcription is resulted from DNA hypermethylation of CpG rich region in *CTH* promoter [28, 30]. DNA methylation, driven by DNMT1, DNMT3A and DNMT3B, can inhibit gene expression. DNMT1 has high affinity for hemi methylated DNA and maintain the constitutive methylation status of DNA [54]. DNMT3A and DNMT3B act primarily as de novo methyltransferases to constitute DNA methylation [55]. Our study indicated that the CpG island of *CTH* promoter was highly methylated and the expression of DNMT3B was markedly increased in FOXC1-overexpressing HCC cells (Huh7-FOXC1) than the control groups. In contrast, the expression of DNMT3B was downregulated and the DNA hypermethylation of *CTH* promoter was inhibited in FOXC1-knockdown cells (MHCC97H-shFOXC1) as compared to control cells. Interestingly, FOXC1 upregulated DNMT3B expression through directly binding to its promoter and transactivated its promoter activities. Moreover, downregulation of DNMT3B decreased FOXC1-mediated HCC proliferation and metastasis, whereas upregulation of DNMT3B reversed the inhibition of HCC proliferation and metastasis caused by FOXC1 down-regulated. In human HCC tissues, FOXC1 expression was positively associated with DNMT3B expression, and the patients with positive co-expression of FOXC1 and DNMT3B had the worst prognosis. These studies indicated that overexpression of FOXC1 induced the DNA hypermethylation of *CTH* promoter and *CTH* gene silencing through upregulating DNMT3B expression, which resulted in HCC proliferation and metastasis.

Although we identified FOXC1 altered the cysteine metabolism and ROS levels, and FOXC1-mediated high level of ROS promoted HCC proliferation and metastasis, the mechanism underlying FOXC1 overexpression in HCC needs to be clarified. In this study, we observed that high level ROS upregulated FOXC1 expression via the ERK1/2-pELK1 pathway in HCC cells. Overexpression of FOXC1 increased ROS levels through regulating cysteine metabolism, which formed a positive feedback loop to facilitate HCC progression. Our in vitro study showed that the antioxidant NAC inhibited ROS-mediated FOXC1 upregulation, thereby inhibiting ROS-FOXC1-cysteine-ROS signaling-mediated HCC proliferation and invasion. Furthermore, FOXC1 expression was positively correlated with 8-OHdG (oxidative damage marker) and p-ELK1 (activated ELK1) expression in human HCC tissues. HCC patients with positive coexpression of 8-OHdG/FOXC1 or p-ELK1/FOXC1 exhibited the worst prognosis. These studies indicated that ROS-ERK1/2-p-ELK1 signaling mediated FOXC1 overexpression promoted HCC progression.

## Conclusions

**Fig. 7:**
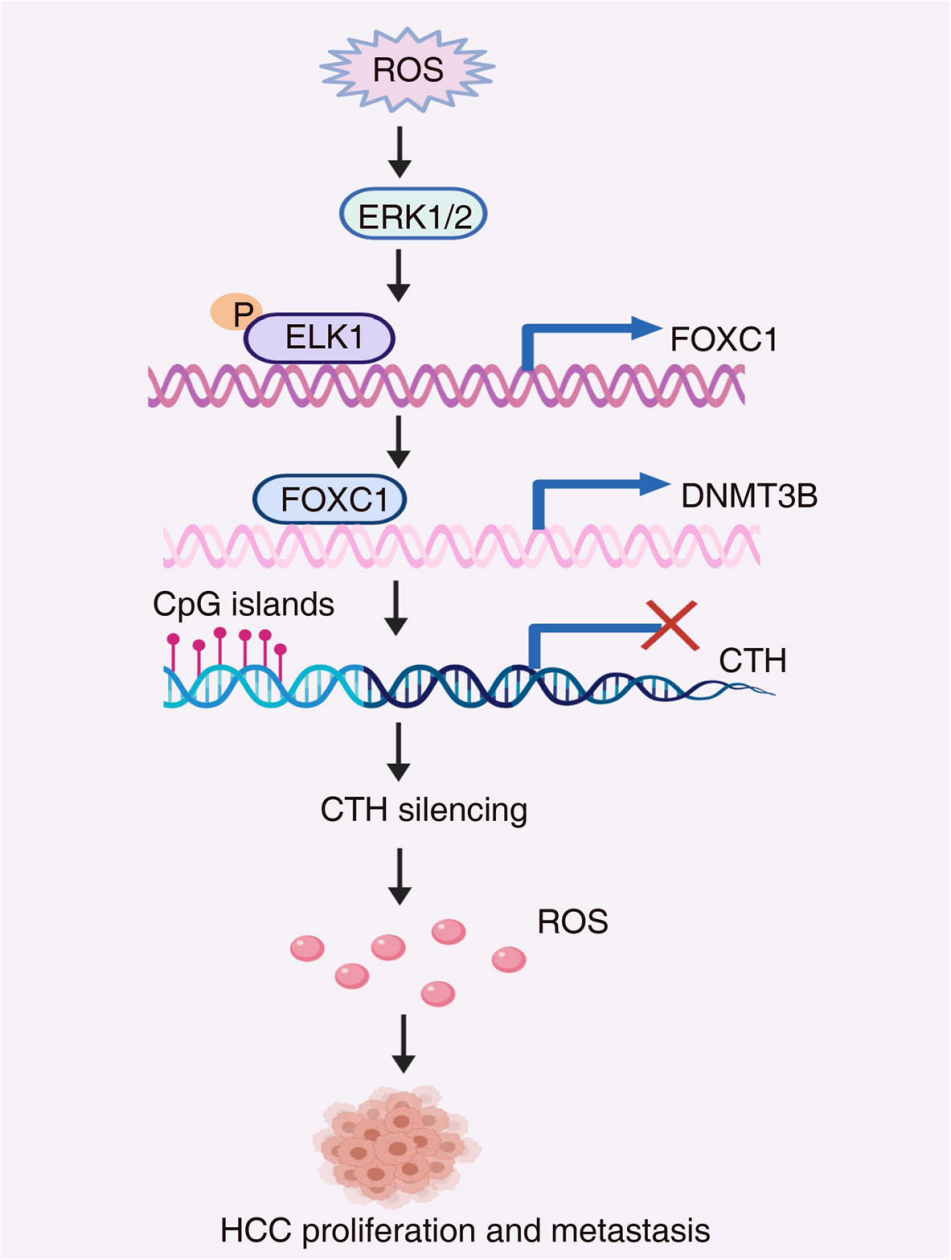
Schematic diagram. A schematic diagram of the role of ROS-FOXC1-cysteine-ROS positive feedback loop in HCC proliferation and metastasis. ROS upregulates FOXC1 expression through the ERK1/2-p-ELK1 signaling pathway. High-expression of FOXC1 induced the DNA hypermethylation of *CTH* promoter and *CTH* gene silencing through upregulating DNMT3B expression, which resulted in the decrease of cysteine levels and increases of ROS levels. The antioxidant NAC inhibits ROS-mediated FOXC1 upregulation, thereby inhibiting ROS-FOXC1-cysteine-ROS signaling-mediated HCC proliferation, migration and invasion

In conclusion, we found the ROS-ERK1/2-p-ELK1 signaling axis upregulated FOXC1 expression in HCC cells. Overexpression of FOXC1 induced the DNA hypermethylation of *CTH* promoter and *CTH* gene silencing through upregulating DNMT3B expression, which resulted in the decrease of cysteine levels and increases of ROS levels (Fig. 7). Thus, we demonstrated that the positive feedback loop of OS-FOXC1-cysteine metabolism-ROS is important for promoting liver cancer proliferation and metastasis, and this pathway may provide a prospective clinical treatment approach for HCC.

## Supplementary Information

**Additional file 1: Supplementary Table S1.** List of genes differentially expressed in Huh7-FOXC1 versus Huh7-control cells using a human amino acid metabolism PCR array. **Supplementary Table S2.** List of genes differentially expressed in MHCC97H-shFOXC1 versus MHCC97H-shcontrol cells using a human amino acid metabolism PCR array. **Supplementary Table S3.** Correlation between CTH methylation and clinicopathological characteristics of HCCs in two independent cohorts of human HCC tissues. **Supplementary Table S4.** Correlation between DNMT3B expression and clinicopathological characteristics of HCCs in two independent cohorts of human HCC tissues. **Supplementary Table S5.** Correlation between 8-OHdG expression and clinicopathological characteristics of HCCs in two independent cohorts of human HCC tissues. **Supplementary Table S6.** Correlation between pELK1 expression and clinicopathological characteristics of HCCs in two independent cohorts of human HCC tissues. **Supplementary Table S7.** Primer sequences used in the study. **Supplementary Table S8.** Knockdown shRNA sequences used in this study.

**Additional file 2: Supplementary Figure S1, Supplementary Figure S2, Supplementary Figure S3, Supplementary Figure S4, Supplementary Figure S5.**

## Data Availability

The data supporting our conclusion were obtained from the TCGA database (https://cancergenome.nih.gov) and Human Protein Atlas online database (https://www.proteinatlas.org).

## Abbreviations

BGS: Bisulfite genomic sequencing
BSO: L-Buthionine-sulfoximine
ChIP: Chromatin immunoprecipitation analysis
CTH: Cystathionine γ-lyase
DNMTs: DNA methylases
FOXC1: Forkhead box C1
HCC: Hepatocellular carcinoma
MSP: Methylation-specific PCR
NAC: N-acetylcysteine
pELK1: Phospho-ETS Transcription Factor 1
ROS: Reactive oxygen species
TNM: Tumor-node-metastasis
5-Aza: 5-aza-2′-deoxycytidine
8-OhdG: 8-hydroxy-2′-deoxyguanosine
