# Supplementary material for: FOXC1 promotes HCC proliferation and metastasis by Upregulating DNMT3B to induce DNA Hypermethylation of CTH promoter
Source: J Exp Clin Cancer Res. 2021 Feb 1;40:50. doi: 10.1186/s13046-021-01829-6 (PMC7852227; doi:10.1186/s13046-021-01829-6)
Supplement: Supplementary file 1 — Additional file 1: Supplementary Table S1. List of genes differentially expressed in Huh7-FOXC1 versus Huh7-control cells using a human amino acid metabolism PCR array. Supplementary Table S2. List of genes differentially expressed in MHCC97H-shFOXC1 versus MHCC97H-shcontrol cells using a human amino acid metabolism PCR array. Supplementary Table S3. Correlation between CTH methylation and clinicopathological characteristics of HCCs in two independent cohorts of human HCC tissues. Supplementary Table S4. Correlation between DNMT3B expression and clinicopathological characteristics of HCCs in two independent cohorts of human HCC tissues. Supplementary Table S5. Correlation between 8-OHdG expression and clinicopathological characteristics of HCCs in two independent cohorts of human HCC tissues. Supplementary Table S6. Correlation between pELK1 expression and clinicopathological characteristics of HCCs in two independent cohorts of human HCC tissues. Supplementary Table S7. Primer sequences used in the study. Supplementary Table S8. Knockdown shRNA sequences used in this study. [file 13046_2021_1829_MOESM1_ESM.zip › Supplementary Table S1.docx]

Supplementary Table S1. List of genes differentially expressed in Huh7-FOXC1 versus Huh7-control cells using a human amino acid metabolism PCR array

| **Symbol** | **Fold change** | **Description** |
| --- | --- | --- |
| APIP | 6.675311823 | APAF1 interacting protein |
| AHCY | 5.934102582 | Adenosylhomocysteinase |
| TAT | 4.926548622 | Tyrosine aminotransferase |
| GLUD1 | 4.891720967 | Glutamate dehydrogenase 1 |
| GPT | 4.856326832 | Glutamic-pyruvate transaminase (alanine aminotransferase) |
| CAD | 4.832618414 | Carbamoyl-phosphate synthetase 2, aspartate transcarbamylase, and dihydroorotase |
| MIF | 4.753135859 | Macrophage migration inhibitory factor (glycosylation-inhibiting factor) |
| ADSL | 4.720031753 | Adenylosuccinate lyase |
| MAOB | 4.684233293 | Monoamine oxidase B |
| AGMAT | 4.617256441 | Adenosylhomocysteinase |
| HDC | 4.572916654 | Histidine decarboxylase |
| MAOA | 4.518346728 | Monoamine oxidase A |
| WBSCR22 | 4.478882358 | Williams Beuren syndrome chromosome region 22 |
| BCAT1 | 4.437532263 | Branched chain amino-acid transaminase 1, cytosolic |
| ACADS | 4.374973283 | Acyl-CoA dehydrogenase, C-2 to C-3 short chain |
| DLD | 4.334956908 | Dihydrolipoamide dehydrogenase |
| PHGDH | 4.329246273 | Phosphoglycerate dehydrogenase |
| SHMT2 | 4.296228542 | Serine hydroxymethyltransferase 2 (mitochondrial) |
| PSAT1 | 4.277054518 | Phosphoserine aminotransferase 1 |
| HIBADH | 4.149056872 | 3-hydroxyisobutyrate dehydrogenase |
| HIBCH | 4.087634553 | 3-hydroxyisobutyryl-CoA hydrolase |
| IDO1 | 4.035980562 | Indoleamine 2,3-dioxygenase 1 |
| KMO | 4.014637548 | Kynurenine 3-monooxygenase (kynurenine 3-hydroxylase) |
| SRR | 3.972782175 | Serine racemase |
| GOT2 | 3.966516048 | Glutamic-oxaloacetic transaminase 2, mitochondrial (aspartate aminotransferase2) |
| GFPT1 | 3.946500167 | Glutamine--fructose-6-phosphate transaminase 1 |
| GLS | 3.928064842 | Glutaminase |
| PPAT | 3.891864135 | Phosphoribosyl pyrophosphate amidotransferase |
| ENOPH1 | 3.851184694 | Enolase-phosphatase 1 |
| GOT1 | 3.835461775 | Glutamic-oxaloacetic transaminase 1, soluble (aspartate aminotransferase 1) |
| ASNS | 3.804434254 | Asparagine synthetase (glutamine-hydrolyzing) |
| PRDX6 | 3.751733194 | Peroxiredoxin 6 |
| ALDH18A1 | 3.749078757 | Aldehyde dehydrogenase 18 family, member A1 |
| ALDH4A1 | 3.709743964 | Aldehyde dehydrogenase 4 family, member A1 |
| CPS1 | 3.665235554 | Carbamoyl-phosphate synthase 1, mitochondrial |
| GAMT | 3.614827286 | Guanidinoacetate N-methyltransferase |
| OAT | 3.587105483 | Ornithine aminotransferase |
| AASDHPPT | 3.567292027 | Aminoadipate-semialdehyde dehydrogenase-phosphopantetheinyl transferase |
| ASH1L | 3.502859592 | Ash1 (absent, small, or homeotic)-like (Drosophila) |
| GCDH | 3.426751890 | Glutaryl-CoA dehydrogenase |
| PLOD3 | 3.348644157 | Procollagen-lysine, 2-oxoglutarate 5-dioxygenase 3 |
| AMD1 | 3.221462805 | Adenosylmethionine decarboxylase 1 |
| GATM | 2.904036979 | Glycine amidinotransferase (L-arginine:glycine amidinotransferase) |
| VARS2 | 2.893627243 | Valyl-tRNA synthetase 2, mitochondrial (putative) |
| TPH2 | 2.870480374 | Tryptophan hydroxylase 2 |
| PYCRL | 2.840673454 | Pyrroline-5-carboxylate reductase-like |
| PYCR1 | 2.820683796 | Pyrroline-5-carboxylate reductase 1 |
| PSPH | 2.700497325 | Phosphoserine phosphatase |
| GNMT | 2.646938541 | Glycine N-methyltransferase |
| TDO2 | 2.487079131 | Tryptophan 2,3-dioxygenase |
| PRODH | 2.398153126 | Proline dehydrogenase (oxidase) 1 |
| ODC1 | 2.330146594 | Ornithine decarboxylase 1 |
| LAP3 | 2.314269151 | Leucine aminopeptidase 3 |
| DNMT1 | 2.257321313 | DNMT1 DNA (cytosine-5-)-methyltransferase 1 |
| HMGCS1 | 2.195594432 | 3-hydroxy-3-methylglutaryl-CoA synthase 1 (soluble) |
| P4HA1 | 2.169520883 | Prolyl 4-hydroxylase, alpha polypeptide I |
| OXCT2 | 2.115917487 | 3-oxoacid CoA transferase 2 |
| EHHADH | 2.053983951 | Enoyl-CoA, hydratase/3-hydroxyacyl CoA dehydrogenase |
| TYR | 2.024252893 | Tyrosinase (oculocutaneous albinism IA) |
| PNMT | 2.001004615 | Phenylethanolamine N-methyltransferase |
| AUH | 1.801449318 | AU RNA binding protein/enoyl-CoA hydratase |
| BCAT2 | 1.705090745 | Branched chain amino-acid transaminase 2, mitochondrial |
| ACAT1 | 1.670930053 | Acetyl-CoA acetyltransferase 1 |
| LARS | 1.560496321 | Leucyl-tRNA synthetase |
| TYRP1 | 1.516051325 | Tyrosinase-related protein 1 |
| MCCC2 | 1.503322316 | Methylcrotonoyl-CoA carboxylase 2 (beta) |
| TH | 1.484097832 | Tyrosine hydroxylase |
| COMT | 1.380176695 | Catechol-O-methyltransferase |
| MUT | 1.230802066 | Methylmalonyl CoA mutase |
| ALDH5A1 | 1.219765713 | Aldehyde dehydrogenase 5 family, member A1 |
| ADSS | 1.206035316 | Adenylosuccinate synthase |
| MTR | 1.174990432 | 5-methyltetrahydrofolate-homocysteine methyltransferase |
| NIT2 | 1.112098623 | Nitrilase family, member 2 |
| AOC3 | 1.080293074 | Amine oxidase, copper containing 3 (vascular adhesion protein 1) |
| ALAS1 | 1.065572785 | Aminolevulinate, delta-, synthase 1 |
| GLDC | 1.062781078 | Glycine dehydrogenase (decarboxylating) |
| ACADSB | 1.061508622 | Acyl-CoA dehydrogenase, short/branched chain |
| ACAT2 | 1.052168761 | Acetyl-CoA acetyltransferase 2 |
| PCCA | 1.043486409 | Propionyl CoA carboxylase, alpha polypeptide |
| BCKDHB | 1.024253896 | Branched chain keto acid dehydrogenase E1, beta polypeptide |
| LDHA | 1.022987256 | Lactate dehydrogenase A |
| MCEE | 1.020969542 | Methylmalonyl CoA epimerase |
| AMT | 1.019102824 | Aminomethyltransferase |
| CAT | 1.016200418 | Catalase |
| CYP1B1 | 1.014321012 | Cytochrome P450, family 1, subfamily B, polypeptide 1 |
| BCKDHA | 1.000570864 | Branched chain keto acid dehydrogenase E1, alpha polypeptide |
| TPO | -1.047018772 | Thyroid peroxidase |
| DBH | -1.086033149 | Dopamine beta-hydroxylase (dopamine beta-monooxygenase) |
| HGD | -1.106058713 | Homogentisate 1,2-dioxygenase |
| AASS | -1.108580826 | Aminoadipate-semialdehyde synthase |
| DLST | -1.148526995 | Dihydrolipoamide S-succinyltransferase component of 2-oxo-glutarate complex) |
| AADAT | -1.156920873 | Aminoadipate aminotransferase |
| OTC | -1.174680847 | Ornithine carbamoyltransferase |
| NOS2 | -1.176992023 | Nitric oxide synthase 2, inducible/Inos |
| TMLHE | -1.185213081 | Trimethyllysine hydroxylase, epsilon |
| NAGS | -1.189682472 | N-acetylglutamate synthase |
| PIPOX | -1.191515643 | Pipecolic acid oxidase |
| ARG2 | -1.194097825 | Arginase, type II |
| BBOX1 | -1.201315124 | Butyrobetaine (gamma), 2-oxoglutarate dioxygenase (gamma-butyrobetaine hydroxylase) 1 |
| OGDH | -1.204239569 | Oxoglutarate (alpha-ketoglutarate) dehydrogenase (lipoamide) |
| HNMT | -1.215216763 | Histamine N-methyltransferase |
| AMDHD1 | -1.219424908 | Amidohydrolase domain containing 1 |
| FTCD | -1.223709713 | Formiminotransferase cyclodeaminase |
| ABP1 | -1.226968532 | Amiloride binding protein 1 (amine oxidase (copper-containing)) |
| PDHA2 | -1.246861682 | Pyruvate dehydrogenase (lipoamide) alpha 2 |
| ALDH3B1 | -1.285255706 | Aldehyde dehydrogenase 3 family, member B1 |
| IARS | -1.289381345 | Isoleucyl-tRNA synthetase |
| ­­DBT | -1.293234905 | Dihydrolipoamide branched chain transacylase E2 |
| HSD17B10 | -1.298919732 | Hydroxysteroid (17-beta) dehydrogenase 10 |
| ASL | -1.313820581 | Argininosuccinate lyase |
| CKB | -1.319339058 | Creatine kinase, brain |
| ALDH9A1 | -1. 321125928 | Aldehyde dehydrogenase 9 family, member A1 |
| ASS1 | -1.323391955 | Argininosuccinate synthase 1 |
| PAH | -1.328161924 | Phenylalanine hydroxylase |
| HPD | -1.346075472 | 4-hydroxyphenylpyruvate dioxygenase |
| CHDH | -1.358808913 | Choline dehydrogenase |
| WARS | -1.359273631 | Tryptophanyl-tRNA synthetase |
| KYNU | -1.370214687 | Kynureninase |
| DDC | -1.371245259 | Dopa decarboxylase (aromatic L-amino acid decarboxylase) |
| DMGDH | -1.379742424 | Dimethylglycine dehydrogenase |
| GCAT | -1.395496842 | Glycine C-acetyltransferase |
| ALDH6A1 | -1.399293226 | Aldehyde dehydrogenase 6 family, member A1 |
| CNDP1 | -1.404813106 | Carnosine dipeptidase 1 (metallopeptidase M20 family) |
| HADHB | -1.408310264 | Hydroxyacyl-CoA dehydrogenase/3-ketoacyl-CoA thiolase/enoyl-CoA hydratase (trifunctional protein), beta subunit |
| ALDH2 | -1.414243912 | Aldehyde dehydrogenase 2 family (mitochondrial) |
| HADH | -1.419154386 | Hydroxyacyl-CoA dehydrogenase |
| ACADM | -1.439504215 | Acyl-CoA dehydrogenase, C-4 to C-12 straight chain |
| SARDH | -1.454086501 | Sarcosine dehydrogenase |
| AANAT | -1.486733405 | Aralkylamine N-acetyltransferase |
| ABAT | -1.556543754 | 4-aminobutyrate aminotransferase |
| FAH | -1.610403775 | Fumarylacetoacetate hydrolase (fumarylacetoacetase) |
| DAO | -1.676553226 | D-amino-acid oxidase |
| BHMT | -1.698183622 | Betaine--homocysteine S-methyltransferase |
| INMT | -1.718540129 | Indolethylamine N-methyltransferase |
| OGDHL | -1.786669657 | Oxoglutarate dehydrogenase-like |
| ACMSD | -1.860304092 | Aminocarboxymuconate semialdehyde decarboxylase |
| PRODH2 | -1.917487253 | Proline dehydrogenase (oxidase) 2 |
| AOX1 | -2.044124926 | Aldehyde oxidase 1 |
| IVD | -2.078767374 | Isovaleryl-CoA dehydrogenase |
| PDHB | -2.130614023 | Pyruvate dehydrogenase (lipoamide) beta |
| ADH5 | -2.192274751 | Alcohol dehydrogenase 5 (class III), chi polypeptide |
| ECHS1 | -2.271392425 | Enoyl CoA hydratase, short chain, 1, mitochondrial |
| SRM | -2.290852147 | Spermidine synthase |
| ACY1 | -2.363183546 | Aminoacylase 1 |
| ADI1 | -2.402248192 | Acireductone dioxygenase 1 |
| MPST | -2. 437327481 | Mercaptopyruvate sulfurtransferase |
| HMGCL | -2.507986213 | 3-hydroxymethyl-3-methylglutaryl-CoA lyase |
| HAAO | -2. 746562418 | 3-hydroxyanthranilate 3,4-dioxygenase |
| SAT1 | -2.766567412 | Spermidine/spermine N1-acetyltransferase 1 |
| MTAP | -2.876394642 | Methylthioadenosine phosphorylase |
| MAT1A | -2. 978871961 | Methionine adenosyltransferase I, alpha |
| ASPA | -3.083561952 | Aspartoacylase |
| GAD2 | -3. 217658164 | Glutamate decarboxylase 2 (pancreatic islets and brain, 65kDa) |
| AGXT | -3.229220041 | Alanine-glyoxylate aminotransferase |
| SDS | -3.270963117 | Serine dehydratase |
| CBS | -3. 428292449 | Cystathionine-beta-synthase |
| CDO1 | -3.478908425 | Cysteine dioxygenase, type I |
| CTH | -4.244924568 | Cystathionase (cystathionine gamma-lyase) |
