# Supplementary material for: FOXC1 promotes HCC proliferation and metastasis by Upregulating DNMT3B to induce DNA Hypermethylation of CTH promoter
Source: J Exp Clin Cancer Res. 2021 Feb 1;40:50. doi: 10.1186/s13046-021-01829-6 (PMC7852227; doi:10.1186/s13046-021-01829-6)
Supplement: Supplementary file 1 — Additional file 1: Supplementary Table S1. List of genes differentially expressed in Huh7-FOXC1 versus Huh7-control cells using a human amino acid metabolism PCR array. Supplementary Table S2. List of genes differentially expressed in MHCC97H-shFOXC1 versus MHCC97H-shcontrol cells using a human amino acid metabolism PCR array. Supplementary Table S3. Correlation between CTH methylation and clinicopathological characteristics of HCCs in two independent cohorts of human HCC tissues. Supplementary Table S4. Correlation between DNMT3B expression and clinicopathological characteristics of HCCs in two independent cohorts of human HCC tissues. Supplementary Table S5. Correlation between 8-OHdG expression and clinicopathological characteristics of HCCs in two independent cohorts of human HCC tissues. Supplementary Table S6. Correlation between pELK1 expression and clinicopathological characteristics of HCCs in two independent cohorts of human HCC tissues. Supplementary Table S7. Primer sequences used in the study. Supplementary Table S8. Knockdown shRNA sequences used in this study. [file 13046_2021_1829_MOESM1_ESM.zip › Supplementary Table S2.docx]

Supplementary Table S2. List of genes differentially expressed in MHCC97H-shFOXC1 versus MHCC97H-shcontrol cells using a human amino acid metabolism PCR array

| **Symbol** | **Fold change** | **Description** |
| --- | --- | --- |
| CTH | 3.924564528 | Cystathionase (cystathionine gamma-lyase) |
| CBS | 3.328249634 | Cystathionine-beta-synthase |
| MTAP | 3.076346429 | Methylthioadenosine phosphorylase |
| ACY1 | 3.031843546 | Aminoacylase 1 |
| FAH | 3.001308775 | Fumarylacetoacetate hydrolase (fumarylacetoacetase) |
| ACMSD | 2.953092572 | Aminocarboxymuconate semialdehyde decarboxylase |
| PDHA2 | 2.923183546 | Pyruvate dehydrogenase (lipoamide) alpha 2 |
| CDO1 | 2.880308775 | Cysteine dioxygenase, type I |
| CKB | 2.853092572 | Creatine kinase, brain |
| MAT1A | 2.816682408 | Methionine adenosyltransferase I, alpha |
| ALDH6A1 | 2.808842905 | Aldehyde dehydrogenase 6 family, member A1 |
| BHMT | 2.764390584 | Betaine--homocysteine S-methyltransferase |
| ABAT | 2.730942572 | 4-aminobutyrate aminotransferase |
| GAD2 | 2.706682408 | Glutamate decarboxylase 2 (pancreatic islets and brain, 65kDa) |
| ACADM | 2.668842905 | Acyl-CoA dehydrogenase, C-4 to C-12 straight chain |
| CHDH | 2.630926572 | Choline dehydrogenase |
| HAAO | 2.566824508 | 3-hydroxyanthranilate 3,4-dioxygenase |
| HADHB | 2.558842905 | Hydroxyacyl-CoA dehydrogenase/3-ketoacyl-CoA thiolase/enoyl-CoA hydratase (trifunctional protein), beta subunit |
| SAT1 | 2.530492572 | Spermidine/spermine N1-acetyltransferase 1 |
| AADAT | 2.526682408 | Aminoadipate aminotransferase |
| HSD17B10 | 2.508842905 | Hydroxysteroid (17-beta) dehydrogenase 10 |
| ASS1 | 2.464390584 | Argininosuccinate synthase 1 |
| AANAT | 2.430421595 | Aralkylamine N-acetyltransferase |
| TPO | 2.290196314 | Thyroid peroxidase |
| OGDH | 2.223681823 | Oxoglutarate (alpha-ketoglutarate) dehydrogenase (lipoamide) |
| NAGS | 2.150114088 | N-acetylglutamate synthase |
| DDC | 2.137565854 | Dopa decarboxylase (aromatic L-amino acid decarboxylase) |
| DAO | 2.117166584 | D-amino-acid oxidase |
| MPST | 2.071232539 | Mercaptopyruvate sulfurtransferase |
| INMT | 2.046418562 | Indolethylamine N-methyltransferase |
| ASPA | 1.981831644 | Aspartoacylase |
| SRM | 1.960651242 | Spermidine synthase |
| SDS | 1.956987364 | Serine dehydratase |
| PRODH2 | 1.941973289 | Proline dehydrogenase (oxidase) 2 |
| AOX1 | 1.886217953 | Aldehyde oxidase 1 |
| AGXT | 1.862179342 | Alanine-glyoxylate aminotransferase |
| IVD | 1.830567384 | Isovaleryl-CoA dehydrogenase |
| AMDHD1 | 1.828621793 | Amidohydrolase domain containing 1 |
| ADI1 | 1.783056734 | Acireductone dioxygenase 1 |
| OGDHL | 1.749275124 | Oxoglutarate dehydrogenase-like |
| HMGCL | 1.725236183 | 3-hydroxymethyl-3-methylglutaryl-CoA lyase |
| ECHS1 | 1.709274512 | Enoyl CoA hydratase, short chain, 1, mitochondrial |
| PDHB | 1.694952361 | Pyruvate dehydrogenase (lipoamide) beta |
| ALDH2 | 1.630224825 | Aldehyde dehydrogenase 2 family (mitochondrial) |
| IARS | 1.614245947 | Isoleucyl-tRNA synthetase |
| CNDP1 | 1.579204201 | Carnosine dipeptidase 1 (metallopeptidase M20 family) |
| SARDH | 1.533274816 | Sarcosine dehydrogenase |
| ADH5 | 1.487101285 | Alcohol dehydrogenase 5 (class III), chi polypeptide |
| KYNU | 1.452932267 | Kynureninase |
| HADH | 1.429054721 | Hydroxyacyl-CoA dehydrogenase |
| PAH | 1.383195256 | Phenylalanine hydroxylase |
| GCAT | 1.374853372 | Glycine C-acetyltransferase |
| ARG2 | 1.344926124 | Arginase, type II |
| ­­DBT | 1.306226553 | Dihydrolipoamide branched chain transacylase E2 |
| ALDH9A1 | 1.277387649 | Aldehyde dehydrogenase 9 family, member A1 |
| ABP1 | 1.219424908 | Amiloride binding protein 1 (amine oxidase (copper-containing)) |
| ASL | 1.219424908 | Argininosuccinate lyase |
| DMGDH | 1.189682472 | Dimethylglycine dehydrogenase |
| OTC | 1.166657697 | Ornithine carbamoyltransferase |
| NOS2 | 1.132919335 | Nitric oxide synthase 2, inducible/Inos |
| ALDH3B1 | 1.130880813 | Aldehyde dehydrogenase 3 family, member B1 |
| WARS | 1.116543754 | Tryptophanyl-tRNA synthetase |
| FTCD | 1.104291243 | Formiminotransferase cyclodeaminase |
| PIPOX | 1.089334581 | Pipecolic acid oxidase |
| HPD | 1.044810639 | 4-hydroxyphenylpyruvate dioxygenase |
| AASS | 1.040586501 | Aminoadipate-semialdehyde synthase |
| DBH | 1.037018772 | Dopamine beta-hydroxylase (dopamine beta-monooxygenase) |
| HNMT | 1.015216763 | Histamine N-methyltransferase |
| BCKDHA | 1.010086477 | Branched chain keto acid dehydrogenase E1, alpha polypeptide |
| BBOX1 | 1.001315124 | Butyrobetaine (gamma), 2-oxoglutarate dioxygenase (gamma-butyrobetaine hydroxylase) 1 |
| CYP1B1 | 1.000101942 | Cytochrome P450, family 1, subfamily B, polypeptide 1 |
| DLST | -1.008526995 | Dihydrolipoamide S-succinyltransferase component of 2-oxo-glutarate complex) |
| LDHA | -1.012987256 | Lactate dehydrogenase A |
| BCKDHB | -1.024389625 | Branched chain keto acid dehydrogenase E1, beta polypeptide |
| TMLHE | -1.085213081 | Trimethyllysine hydroxylase, epsilon |
| AOC3 | -1.110293074 | Amine oxidase, copper containing 3 (vascular adhesion protein 1) |
| HGD | -1.116058713 | Homogentisate 1,2-dioxygenase |
| ACADSB | -1.121508622 | Acyl-CoA dehydrogenase, short/branched chain |
| CAT | -1.162004183 | Catalase |
| MUT | -1.230802066 | Methylmalonyl CoA mutase |
| LARS | -1.260632491 | Leucyl-tRNA synthetase |
| PCCA | -1.304348692 | Propionyl CoA carboxylase, alpha polypeptide |
| ADSS | -1.336031536 | Adenylosuccinate synthase |
| AUH | -1.380141894 | AU RNA binding protein/enoyl-CoA hydratase |
| MCCC2 | -1.402231633 | Methylcrotonoyl-CoA carboxylase 2 (beta) |
| PRODH | -1.448152614 | Proline dehydrogenase (oxidase) 1 |
| COMT | -1.480669517 | Catechol-O-methyltransferase |
| EHHADH | -1.513983513 | Enoyl-CoA, hydratase/3-hydroxyacyl CoA dehydrogenase |
| NIT2 | -1.520623982 | Nitrilase family, member 2 |
| P4HA1 | -1.562089583 | Prolyl 4-hydroxylase, alpha polypeptide I |
| MTR | -1.574049932 | 5-methyltetrahydrofolate-homocysteine methyltransferase |
| AMD1 | -1.601462805 | Adenosylmethionine decarboxylase 1 |
| ACAT1 | -1.609300533 | Acetyl-CoA acetyltransferase 1 |
| GLDC | -1.632107886 | Glycine dehydrogenase (decarboxylating) |
| TPH2 | -1.648404374 | Tryptophan hydroxylase 2 |
| AMT | -1.670930053 | Aminomethyltransferase |
| LAP3 | -1.702107886 | Leucine aminopeptidase 3 |
| ALDH18A1 | -1.704840374 | Aldehyde dehydrogenase 18 family, member A1 |
| ALDH4A1 | -1.719228104 | Aldehyde dehydrogenase 4 family, member A1 |
| ALAS1 | -1.742694151 | Aminolevulinate, delta-, synthase 1 |
| HMGCS1 | -1.790475778 | 3-hydroxy-3-methylglutaryl-CoA synthase 1 (soluble) |
| MCEE | -1.809743964 | Methylmalonyl CoA epimerase |
| DNMT1 | -1.865278575 | DNMT1 DNA (cytosine-5-)-methyltransferase 1 |
| ACAT2 | -1.895439425 | Acetyl-CoA acetyltransferase 2 |
| OXCT2 | -1.902095424 | 3-oxoacid CoA transferase 2 |
| OAT | -1.925732113 | Ornithine aminotransferase |
| ALDH5A1 | -1.952168761 | Aldehyde dehydrogenase 5 family, member A1 |
| PYCRL | -1.974073454 | Pyrroline-5-carboxylate reductase-like |
| PYCR1 | -1.978203796 | Pyrroline-5-carboxylate reductase 1 |
| TH | -2.004097832 | Tyrosine hydroxylase |
| GATM | -2.097369739 | Glycine amidinotransferase (L-arginine:glycine amidinotransferase) |
| TYRP1 | -2.116132505 | Tyrosinase-related protein 1 |
| AASDHPPT | -2.167202747 | Aminoadipate-semialdehyde dehydrogenase-phosphopantetheinyl transferase |
| BCAT2 | -2.210907345 | Branched chain amino-acid transaminase 2, mitochondrial |
| PRDX6 | -2.251319473 | Peroxiredoxin 6 |
| SRR | -2.272775821 | Serine racemase |
| GNMT | -2.316193854 | Glycine N-methyltransferase |
| GAMT | -2.334828672 | Guanidinoacetate N-methyltransferase |
| MIF | -2.336135985 | Macrophage migration inhibitory factor (glycosylation-inhibiting factor) |
| ACADS | -2.374283973 | Acyl-CoA dehydrogenase, C-2 to C-3 short chain |
| SHMT2 | -2.390254228 | Serine hydroxymethyltransferase 2 (mitochondrial) |
| HDC | -2.429164654 | Histidine decarboxylase |
| ODC1 | -2.473046032 | Ornithine decarboxylase 1 |
| ENOPH1 | -2.485184614 | Enolase-phosphatase 1 |
| PNMT | -2.501001465 | Phenylethanolamine N-methyltransferase |
| HIBADH | -2.539056072 | 3-hydroxyisobutyrate dehydrogenase |
| HIBCH | -2.557633455 | 3-hydroxyisobutyryl-CoA hydrolase |
| TDO2 | -2.587071391 | Tryptophan 2,3-dioxygenase |
| GOT1 | -2.625477615 | Glutamic-oxaloacetic transaminase 1, soluble (aspartate aminotransferase 1) |
| GCDH | -2.636751089 | Glutaryl-CoA dehydrogenase |
| AGMAT | -2.672564441 | Adenosylhomocysteinase |
| GOT2 | -2.706516048 | Glutamic-oxaloacetic transaminase 2, mitochondrial (aspartate aminotransferase2) |
| GFPT1 | -2.734650167 | Glutamine--fructose-6-phosphate transaminase 1 |
| PSPH | -2.749073025 | Phosphoserine phosphatase |
| TYR | -2.770252893 | Tyrosinase (oculocutaneous albinism IA) |
| WBSCR22 | -2.828882358 | Williams Beuren syndrome chromosome region 22 |
| BCAT1 | -2.837563322 | Branched chain amino-acid transaminase 1, cytosolic |
| GPT | -2.856683232 | Glutamic-pyruvate transaminase (alanine aminotransferase) |
| VARS2 | -2.880621143 | Valyl-tRNA synthetase 2, mitochondrial (putative) |
| CPS1 | -2.905552354 | Carbamoyl-phosphate synthase 1, mitochondrial |
| DLD | -2.914908956 | Dihydrolipoamide dehydrogenase |
| ASNS | -2.940489434 | Asparagine synthetase (glutamine-hydrolyzing) |
| PLOD3 | -2.961445157 | Procollagen-lysine, 2-oxoglutarate 5-dioxygenase 3 |
| AHCY | -2.980102582 | Adenosylhomocysteinase |
| MAOB | -3.004238293 | Monoamine oxidase B |
| IDO1 | -3.035056982 | Indoleamine 2,3-dioxygenase 1 |
| KMO | -3.063753485 | Kynurenine 3-monooxygenase (kynurenine 3-hydroxylase) |
| PPAT | -3.186489135 | Phosphoribosyl pyrophosphate amidotransferase |
| ASH1L | -3.200859592 | Ash1 (absent, small, or homeotic)-like (Drosophila) |
| CAD | -3.281841431 | Carbamoyl-phosphate synthetase 2, aspartate transcarbamylase, and dihydroorotase |
| PSAT1 | -3.327051845 | Phosphoserine aminotransferase 1 |
| APIP | -3.436711823 | APAF1 interacting protein |
| ADSL | -3.600131753 | Adenylosuccinate lyase |
| GLS | -3.678842064 | Glutaminase |
| MAOA | -3.783046728 | Monoamine oxidase A |
| PHGDH | -3.889116273 | Phosphoglycerate dehydrogenase |
| GLUD1 | -3.891967720 | Glutamate dehydrogenase 1 |
| TAT | -4.026622548 | Tyrosine aminotransferase |
