# Supplementary material for: FOXC1 promotes HCC proliferation and metastasis by Upregulating DNMT3B to induce DNA Hypermethylation of CTH promoter
Source: J Exp Clin Cancer Res. 2021 Feb 1;40:50. doi: 10.1186/s13046-021-01829-6 (PMC7852227; doi:10.1186/s13046-021-01829-6)
Supplement: Supplementary file 1 — Additional file 1: Supplementary Table S1. List of genes differentially expressed in Huh7-FOXC1 versus Huh7-control cells using a human amino acid metabolism PCR array. Supplementary Table S2. List of genes differentially expressed in MHCC97H-shFOXC1 versus MHCC97H-shcontrol cells using a human amino acid metabolism PCR array. Supplementary Table S3. Correlation between CTH methylation and clinicopathological characteristics of HCCs in two independent cohorts of human HCC tissues. Supplementary Table S4. Correlation between DNMT3B expression and clinicopathological characteristics of HCCs in two independent cohorts of human HCC tissues. Supplementary Table S5. Correlation between 8-OHdG expression and clinicopathological characteristics of HCCs in two independent cohorts of human HCC tissues. Supplementary Table S6. Correlation between pELK1 expression and clinicopathological characteristics of HCCs in two independent cohorts of human HCC tissues. Supplementary Table S7. Primer sequences used in the study. Supplementary Table S8. Knockdown shRNA sequences used in this study. [file 13046_2021_1829_MOESM1_ESM.zip › Supplementary Table S4.docx]

Supplementary Table S4. Correlation between DNMT3B expression and clinicopathological characteristics of HCCs in two independent cohorts of human HCC tissues

|  |  | Cohort I | |  |  | Cohort II | |  |
| --- | --- | --- | --- | --- | --- | --- | --- | --- |
| Clinicopathological variables | | Tumor DNMT3B expression | | *P* Value |  | Tumor DNMT3B expression | | *P* Value |
|  |  | Negative (n=135) | Positive (n=145) |  |  | Negative (n=107) | Positive (n=103) |  |
| Age | | 52.80(10.114) | 51.62(9.932) | 0.816 |  | 52.34(10.391) | 53.09(10.772) | 0.713 |
| Sex | female | 19 | 26 | 0.418 |  | 15 | 24 | 0.110 |
|  | male | 116 | 119 |  |  | 92 | 79 |  |
| Serum AFP | ≤20ng/ml | 27 | 22 | 0.345 |  | 27 | 23 | 0.631 |
|  | >20ng/ml | 108 | 123 |  |  | 80 | 80 |  |
| Virus infection | HBV | 85 | 108 | 0.151 |  | 86 | 72 | 0.247 |
|  | HCV | 23 | 21 |  |  | 6 | 13 |  |
|  | HBV+HCV | 10 | 6 |  |  | 5 | 5 |  |
|  | none | 17 | 10 |  |  | 10 | 13 |  |
| Cirrrhosis | absent | 39 | 40 | 0.894 |  | 25 | 32 | 0.219 |
|  | present | 96 | 105 |  |  | 82 | 71 |  |
| Child-pugh score | Class A | 117 | 119 | 0.326 |  | 83 | 75 | 0.523 |
|  | Class B | 18 | 26 |  |  | 24 | 28 |  |
| Tumor number | single | 108 | 83 | <0.001 |  | 72 | 52 | 0.017 |
|  | multiple | 27 | 62 |  |  | 35 | 51 |  |
| Maximal tumor size | ≤5cm | 90 | 72 | 0.005 |  | 51 | 48 | 0.891 |
|  | >5cm | 45 | 73 |  |  | 56 | 55 |  |
| Tumor encapsulation | absent | 19 | 56 | <0.001 |  | 29 | 57 | <0.001 |
|  | present | 116 | 89 |  |  | 78 | 46 |  |
| Microvascular invasion | absent | 106 | 66 | <0.001 |  | 74 | 42 | <0.001 |
|  | present | 29 | 79 |  |  | 33 | 61 |  |
| Tumor differentiation | I-II | 121 | 86 | <0.001 |  | 97 | 70 | <0.001 |
|  | III-Ⅳ | 14 | 59 |  |  | 10 | 33 |  |
| TNM stage | I-II | 130 | 93 | <0.001 |  | 101 | 69 | <0.001 |
|  | III | 5 | 52 |  |  | 6 | 34 |  |
