# Supplementary material for: FOXC1 promotes HCC proliferation and metastasis by Upregulating DNMT3B to induce DNA Hypermethylation of CTH promoter
Source: J Exp Clin Cancer Res. 2021 Feb 1;40:50. doi: 10.1186/s13046-021-01829-6 (PMC7852227; doi:10.1186/s13046-021-01829-6)
Supplement: Supplementary file 2 — Additional file 2: Supplementary Figure S1, Supplementary Figure S2, Supplementary Figure S3, Supplementary Figure S4, Supplementary Figure S5. [file 13046_2021_1829_MOESM2_ESM.zip › Supporting Information.docx]

**Supplementary material**

**Methods**

**Cell culture**

Human HCC cells (Huh-7 and PLC/PRF/5) were purchased from the American Type Culture Collection. Additional human HCC cells (MHCC97H) were kindly provided by Dr. Tang ZY (Liver Cancer Institute, Zhongshan Hospital, Fudan University, Shanghai, China). Huh7 and PLC/PRF/5, are HCC cells with low metastatic potential, whereas MHCC97H are HCC cells with high metastatic potential. Cells were cultured in Dulbecco’s Modified Eagle Medium (DMEM) at 37°C in a 5% CO2 incubator. The medium was supplemented with 10% FBS, 100 μg/ml penicillin, and 100 μg/ml streptomycin. These above cell lines were authenticated by short tandem repeats (STRs) DNA profiling. All cells were tested for mycoplasma contamination before use with the Universal Mycoplasma Detection Kit (ATCC 30-1012K) and were not contaminated by mycoplasma.

**Construction of tissue microarrays and immunohistochemistry**

HCC samples and the corresponding adjacent liver tissues were used to construct a tissue microarray (Shanghai Biochip Co., Ltd. Shanghai, China). The tissue microarray was stained for FOXC1(Abcam, ab227977, 1：400), CTH (Proteintech, 12217-1-AP，1：200), DNMT3B (Abcam, ab2851，1：500), 8-OHdG (Abcam, ab48508, 1:200) and phospho-ELK1 (Ser383; Santa Cruz Biotechnology, sc-8406, 1:200) expression. The array was scored independently by two pathologists for both the staining intensity and the extent of the protein expression across the section.

According to the manufacture’s instruction of SAP test kit (Zhongshan jinqiao, China), the tissue sections were baked on a panel at 60 °C for an hour, and then deparaffinized with xylene and rehydrated through gradient ethanol immersion. Then the slides were immersed in 0.01 mol/L citrate buffer solution (pH 6.0) and placed in a microwave oven for 20 min. Endogenous peroxidase activity was quenched by 3% (vol/vol) hydrogen peroxide in methanol for 12 min, followed by three 3-min washes with phosphate-buffered saline (PBS). After washing in PBS, the sections were incubated with bovine serum albumin (BSA) for 30 min, then incubated in a moist chamber at 4 °C overnight with the primary antibody diluted in PBS containing 1% (wt/vol) BSA. Negative controls were performed by replacing the primary antibody with preimmune mouse serum. After three 5 min washes with PBS, the sections were treated with a peroxidase-conjugated second antibody for 30 min at room temperature, followed by additional three 5 min washes with PBS. Reaction product was visualized with diaminobenzidine for 2 min. Images were obtained under a light microscope (Olympus, Japan) equipped with a DP70 digital camera.

Analyses were performed by two independent observers who were blinded to the clinical outcome. The immunostaining intensity was scored on a scale of 0 to 3: 0 (negative), 1 (weak), 2 (medium) or 3 (strong). The percentage of positive cells was evaluated on a scale of 0 to 4: 0 (negative), 1 (1%-25%), 2 (26%-50%), 3 (51%-75%), or 4 (76%-100%). The final immuno-activity scores were calculated by multiplying the above two scores, resulting an overall score which range from 0~12. Each case was ultimately considered “negative” if the final score ranges from 0~3, and “positive” if the final score ranges from 4~12.

A detailed description of the materials and methods used in this study can be found in the online supplementary material.

**Construction of lentivirus and stable cell lines**

Lentiviral vectors encoding shRNAs were generated using PLKO.1-TRC (Addgene) and designated as LV-shFOXC1, LV-shCTH, LV-shDNMT3B, and LV-shControl. “LV-shControl” is a non-target shRNA control. The vector “pLKO.1-puro Non-Target shRNA Control Plasmid DNA” (purchased from Sigma, SHC016) contains an shRNA insert that does not target any known genes from any species. These short hairpin RNAs (shRNAs) sequences were presented in Supplementary Table S8. Lentiviral vectors encoding the human *FOXC1, CTH* and *DNMT3B* genes were constructed in FUW-teto (Addgene) and designated as LV-FOXC1, LV-CTH, and LV-DNMT3B. An empty vector was used as the negative control and was designated as LV-control. Concentrated lentivirus was transfected into the HCC cells with a multiplicity of infection (MOI) ranging from 30 to 50 in the presence of polybrene (6 μg/ml). Seventy-two hours after infection, HCC cells were selected for 2 weeks using 2.5 μg/ml puromycin (OriGene). Selected pools of knockdown and over-expressing cells were used for the following experiments.

**Transient transfection**

The cells were plated at a density of 1×10^5^ cells/well in a 24-well plate. After 12-24 hours, the cells were co-transfected with 0.6 μg of the expression vector plasmids, 0.18 μg of the promoter reporter plasmids, and 0.02 μg of the pRL-TK plasmids using Lipofectamine 3000 (Invitrogen, USA) according to the manufacturer’s instructions. After 8 h of transfection, the cells were washed and allowed to recover overnight in fresh medium supplemented with 1% FBS for 48 h. Serum-starved cells were used for the assay.

**Luciferase reporter assay**

Luciferase activity was detected using the Dual Luciferase Assay (Promega, USA) according to the manufacturer’s instructions. The transfected cells were lysed in culture dishes containing a lysis buffer, and the resulting lysates were centrifuged at maximum speed for 1 min in an Eppendorf microcentrifuge. Relative luciferase activity was determined using a Modulus^TM^ TD20/20 Luminometer (Turner Biosystems, USA), and the transfection efficiencies were normalized according to the Renilla activity.

**Western blot analyses**

Proteins from lysed cells were fractionated by SDS-PAGE and transferred to nitrocellulose membranes. Nonspecific binding sites were blocked with 5% BSA in TBST (120 mM Tris–HCl (pH 7.4), 150 mM NaCl, and 0.05% Tween 20) for 1 hr at room temperature. Blots were incubated with a specific antibody overnight at 4 °C. Western blotting of β-actin on the same membrane was used as a loading control. The membranes were incubated with primary antibodies anti-FOXC1 (Abcam, ab227977,1:1000), anti-CTH(Proteintech, 12217-1-AP,1:2000), anti-DNMT1(Proteintech, 24206-1-AP,1:800)，anti-DNMT3A(Proteintech, 20954-1-AP,1:2000), anti- DNMT3B(Abcam, ab2851,1:1000), anti-ELK1(Proteintech, 27420-1-AP,1:1000), anti-phospho-ELK1 (Ser383; Santa Cruz Biotechnology, sc-8406, 1:1000) and anti-β-actin (Proteintech, 60008-1-lg,1:4000) overnight at 4 °C. The membranes were then washed with TBS-T 3 times and incubated with an HRP-conjugated secondary antibody. Proteins were visualized using an Immobilon^TM^ Western Chemiluminescent HRP substrate (Tanon, China).

**Real-time PCR**

Total RNA was extracted using TRIzol Reagent (TAKARA), and reverse transcription was performed using the Advantage RT-for-PCR Kit (Vazyme) according to the manufacturer’s instructions. For the real-time PCR analyses, aliquots of double-stranded cDNA were amplified using a SYBR Green PCR Kit (Vazyme). The cycling parameters were as follows: 95 °C for 15 s, 55-60 °C for 15 s, and 72 °C for 15 s for 45 cycles. A melting curve analyses was then performed. The Ct was measured during the exponential amplification phase, and the amplification plots were analyzed using SDS 1.9.1 software (Applied Biosystems). For the cell lines, the relative expression levels (defined as the fold change) of the target genes were determined by the following equation: 2^–ΔΔCt^ (ΔCt= ΔCt^target^ – ΔCt^GAPDH^; ΔΔCt = ΔCt^expressing vector^ – ΔCt^control vector^). The expression level was normalized to the fold change that was detected in the corresponding control cells, which was defined as 1.0. For the clinical tissue samples, the fold change of the target gene was determined by the following equation: 2^–ΔΔCt^ (ΔΔCt= ΔCt^tumor^ – ΔCt^nontumor^). This value was normalized to the average fold change in the normal colon epithelial tissues, which was defined as 1.0. All reactions were performed in duplicate. The primer sequences are listed in Supplementary Table S6.

**Measurement of SAMe**.

SAMe levels were determined by liquid chromatography/mass spectrometry (LC/MS) using a Waters Acquity ultraperformance liquid chromatography (UPLC) system equipped with a column oven, and a Waters Acquity UPLC BEH C_18_ column (2.1 ×100 mm, 1.7 μm).A 3-μL aliquot of sample was injected into the UPLC column, which was maintained at 50°C and eluted at 600μL/min. The mobile phase consisted of water with 0.05% formic acid (solvent A) and acetonitrile with 0.05% formic acid (solvent B). Separation was carried out starting with 1% B for 1 minute, followed by a linear gradient from 1% to 95% B for 10 minutes and staying at 95% B for 6 minutes. The UPLC system was coupled to a Waters Micromass LCT Premier Mass Spectrometer equipped with a Lockspray ionization source operating in electrospray positive ion mode (W mode, resolution 10,000 full width at half maximum). Full-scan time-of-flight MS spectra were acquired between m/z (mass-to-charge ratio) 80 and 1,000 using the dynamic range enhancement mode, with a total scan time of 0.4 seconds. Leucine enkephalin was used as a reference compound for accurate mass measurements. The reference was infused into the Lockspray reference channel, and the masses of the analyte channel were automatically mass-corrected by the software. A set of samples, prepared from all standard compounds, was acquired over the concentration range of 10 pg/μLto50ng/μL in order to test the linearity of the system. After analyzing all concentration levels, calibration curves were generatedμL and chromatogram traces of the protonated species of the standards were extracted with a mass window of 0.05 Da. SAMe levels were automatically calculated from the calibration curves.

**Chromatin immunoprecipitation Assay (ChIP)**

Cells were cross-linked in 1% formaldehyde at 37 °C for 10 min. Glycine was added to the final concentration of 0.125 M to terminate the cross-linking, and then were mixed and placed at room temperature for 5 minutes. After washing with PBS, the cells were resuspended in 300 μl of lysis buffer. The DNA was sheared to small fragments by sonication. Sonicated chromatin was diluted to a final SDS concentration of 0.1% and aliquots were rotated with antibody O/N at 4 °C. The recovered supernatants were incubated with specific antibodies or an isotype control IgG for 2 hours in the presence of herring sperm DNA and Protein A/G Magnetic beads (Thermo Fisher). These antibodies are anti-FOXC1(Abcam, ab5079), and anti-ELK1(Abcam, ab32106). The immunoprecipitated DNA was retrieved from the beads with 1% SDS and a 1.1 M NaHCO3 solution at 65 °C for 6 hr. The DNA was then purified using a PCR Purification Kit (QIAGEN, USA). The primers are shown in Supplementary Table S6.

For ChIP assays of tissues, cells were first separated from six fresh frozen HCC tissues and three normal liver tissues collected after surgical resection. In detail, surgically extracted tumor tissues were first washed by 1×cold, PBS, 5min, for three times and added to medium supplemented with antibiotic and antifungal agents. Use a clean razor blade to cut a pie of tissue (around 5mm3) into small piece (typical 1mm3 or smaller). Then, digestion the tissues with DNase I (20 mg/mL; Sigma-Aldrich) and collagenase (1.5 mg/mL; Sigma-Aldrich) and placed on table concentrator, 37℃, for 1h. At the end of the hour, we filtered the dissociated cells through 100-μm-pore filters rinsed with fresh media. The 1×red cell lysis was added to the tissues and incubated for 5 minutes to lysis the red blood cell, followed by another rinse. The dissociated cells were crosslinked using 1% formaldehyde for 10 minutes at 37℃. Glycine was added to the final concentration of 0.125 M to terminate the cross-linking, and then were mixed and placed at room temperature for 5 minutes. After cell lysis, the DNA was fragmented by sonication. ChIP grade antibody anti-FOXC1(Abcam, ab5079), or IgG (negative control) were used to immunoprecipitated the fragment DNA. Then, qRT-PCR was used to amplify the corresponding binding site on the promoters.

***In vitro* migration and invasion assays**

A 24-well transwell plate (8-µm pore size, Corning, USA) was used to measure the migratory and invasion capacity of each tested cell line. For transwell migration assays, 5×10^4^ cells were plated in the top chamber lined with a non-coated membrane. For invasion assays, chamber inserts were coated with 200 mg/ml of Matrigel and dried overnight under sterile conditions. Then, 1×10^5^ cells were plated in the top chamber. The mean of triplicate assays for each experimental condition was used. The average number of cells in five fields per membrane was counted in triplicate inserts. The relative invasion/migration was expressed as the number of treated cells to control cells.

**CCK8, Colony formation assay, and Soft agar colony formation assay**

The proliferation of HCC cells in vitro was measured using the CCK8 assay. 5000 stably infected cells were seeded into each well of 96‐well plates. Six wells of each group were detected every day. 100 μl fresh medium containing 10μl CCK8 was put into each cell and incubated at 37°C for 2hrs, then the medium was replaced by 100 μl of DMSO and shaken at room temperature for 10 mins. The absorbance was measured at 450 nm.

For colony formation assays, 500 cells were seeded into 35 mm dishes and shaked up. Then the cells were incubated at 37°C in a humidified atmosphere containing 5% CO2 in air for 2 weeks. Subsequently, we removed the medium and stained the cells with crystal violet, captured the dishes with a camera. Only positive colonies (diameter > 40 um) in the dishes were counted and compared.

**Cell cycle analysis**

We seeded 4 x 10^5 cells per well in a six-well plate. Next day, the serum was removed overnight in order to synchronize the cell cycle stage. We changed with fresh medium with serum, and the cells were collected. The cells were fixed with 70% ethanol and then added with 0.5% Triton X-100 and 0.05% RNase at 37℃ for 1 hour. The cells were treated with 50 µg/mL propidium-iodide at 4℃ for 20 minutes followed by detection of the cell cycle using flow cytometry.

***In vivo* tumor growth assay**

For the xenograft tumor growth assay, 5×10^4^ cells were injected subcutaneously into the right flank of 5-week-old male BALB/C nude mice. Each group consisted of 10 mice. Tumor formation in nude mice was monitored over a 30-day period, and the tumor volume was measured every 5 days and calculated as: 1/2 (largest diameter) × (smallest diameter)^2^.

**Quantification and Statistical Analysis**

The quantitative data were compared between groups using the Student's t-test. Categorical data were analyzed using the Fisher’s exact test. The cumulative recurrence and survival rates were determined using the Kaplan-Meier method and log-rank test. The Cox proportional hazards model was used to determine the independent factors that influence survival and recurrence based on the variables that had been selected from the univariate analyses. A value of p < 0.05 was considered to be significant. All the analyses were performed using the SPSS software (version 16.0).

**Figure legends：**

**Supplementary Figure S1.**

(A) Representative data obtained from The Cancer Genome Atlas dataset showing relative mRNA expression levels of *CTH* in normal liver versus liver cancer tissues, and the normal tissues versus cholangiocarcinoma (CHOL), kidney renal clear cell carcinoma (KIRC) and thyroid carcinoma (THCA) tissues. Box-and-whisker plots indicate the median (horizontal line), interquartile range (box), and 10th–90th percentiles (whiskers). *P < 0.05.

(B) IHC staining levels for CTH in liver cancer tissues obtained from the Human Protein Atlas database.

(C) Kaplan–Meier analysis of data obtained from The Cancer Genome Atlas database revealed a correlation between *CTH* mRNA expression levels and overall survival in LIHC.

(D) Representative data obtained from The Cancer Genome Atlas dataset showing relative mRNA expression levels of *DNMT3B* in normal liver versus liver cancer tissues Box-and-whisker plots indicate the median (horizontal line), interquartile range (box), and 10th–90th percentiles (whiskers). *P < 0.05.

(E) IHC staining levels for DNMT3B in liver cancer tissues obtained from the Human Protein Atlas database.

(F) Kaplan–Meier analysis of data obtained from The Cancer Genome Atlas database revealed a correlation between *DNMT3B* mRNA expression levels and overall survival (left panel) and disease-free survival (right panel) in LIHC.

**Supplementary Figure S2.**

(A) Schematic diagram of cysteine metabolism and glutathione metabolism.

(B) Quantitative analysis of western blot assays showing FOXC1 and CTH protein levels in the HCC cells with upregulation or downregulation of FOXC1.

(C) Western blot assays showed that FOXC1 could not regulate the protein levels of CBS in HCC cells.

(D) The SAM levels in the HCC cells with upregulation or downregulation of FOXC1.

(E) The level of ROS was detected in the indicated HCC cells by flow cytometry.

(F) Quantitative analysis of western blot assays showing FOXC1 and CTH protein levels in the stable HCC cells.

(G) Soft agar colony formation assay showed the proliferation abilities of the indicated HCC cells.

(H) The cell cycle of indicated HCC cells were analyzed by flow cytometry.

(I) Representative Ki67 staining images of tumors from different groups. Scale bars represent 50μm.

(J) Incidence of lung metastasis after transplantation in nude mice from different groups.

(K) The overall survival time of the indicated groups.

Data are represented as mean ± S.D. of three experiments. *P<0.05.

**Supplementary Figure S3.**

(A) Quantitative analysis of western blot assays showing CTH protein levels in the indicated HCC cells treated with DMSO or 5-Aza.

(B) After the cells were treated with histone deacetylase inhibitor trichostatin A (TSA) (500 ng/ml, 24 hours), western blots were used to detect the protein levels of CTH.

(C) Quantitative analysis of western blot assays showing FOXC1, DNMT1, DNMT3A and DNMT3B protein levels in the HCC cells with upregulation or downregulation of FOXC1.

(D) Quantitative analysis of western blot assays showing DNMT3B and CTH protein levels in the indicated HCC cells.

(E) BGS detected the methylation status of the *CTH* promoter in the indicated HCC cells displayed that DNMT3B ectopic expression was really associated with a gain of CTH promoter methylation. A typical CpG island is presented at the promoter region of *CTH*. Each red vertical bar represents a single CpG site. The transcription starts site (TSS) is indicated by a curved arrow. Black spots represent 75-100% methylated CpGs, yellow spots denote 50-75% methylated CpGs, orange spots denote 25-50% methylated CpGs and hollow dots represent 0-25% methylated CpGs.

Data are represented as mean ± S.D. of three experiments. *P<0.05.

**Supplementary Figure S4.**

(A-B) Soft agar colony formation assay showed the proliferation abilities of the indicated HCC cells.

(C-D) CCK8 assays detected the proliferation capacities of the indicated HCC cells.

(E-F) The cell cycle of indicated HCC cells were analyzed by flow cytometry.

(G) Representative Ki67 staining images of tumors from different groups. Scale bars represent 50μm.

(H Incidence of lung metastasis after transplantation in nude mice from different groups.

(I) The overall survival time of the indicated groups.

Data are represented as mean ± S.D. of three experiments. *P<0.05.

**Supplementary Figure S5.**

(A) The level of ROS was detected in the indicated HCC cells treated with DMSO or NAC by flow cytometry.

(B) The level of ROS was detected in the indicated HCC cells treated with DMSO or BSO by flow cytometry.

(C) The level of ROS was detected in the Huh7 or PLC/PRF/5 cells treated with DMSO or BSO or NAC by flow cytometry.

(D) Quantitative analysis of western blot assays showing FOXC1 protein levels in the HCC cells treated with DMSO or BSO or NAC.

(E) Quantitative analysis of western blot assays showing FOXC1, ELK1 and Pelk1 protein levels in Huh7 cells treated with BSO and then transfected with lentivirus LV-shELK1 to inhibit the expression of ELK1.

(F) Quantitative analysis of western blot assays showing the protein levels of total and phosphorylated Akt, ERK1/2, JNK, p38 and p65 and FOXC1 expression upon BSO stimulation and indicated inhibitor treatment.

Data are represented as mean ± S.D. of three experiments. *P<0.05.
