# Supplementary figures and images for: FOXC1 promotes HCC proliferation and metastasis by Upregulating DNMT3B to induce DNA Hypermethylation of CTH promoter
Source: J Exp Clin Cancer Res. 2021 Feb 1;40:50. doi: 10.1186/s13046-021-01829-6 (PMC7852227; doi:10.1186/s13046-021-01829-6)

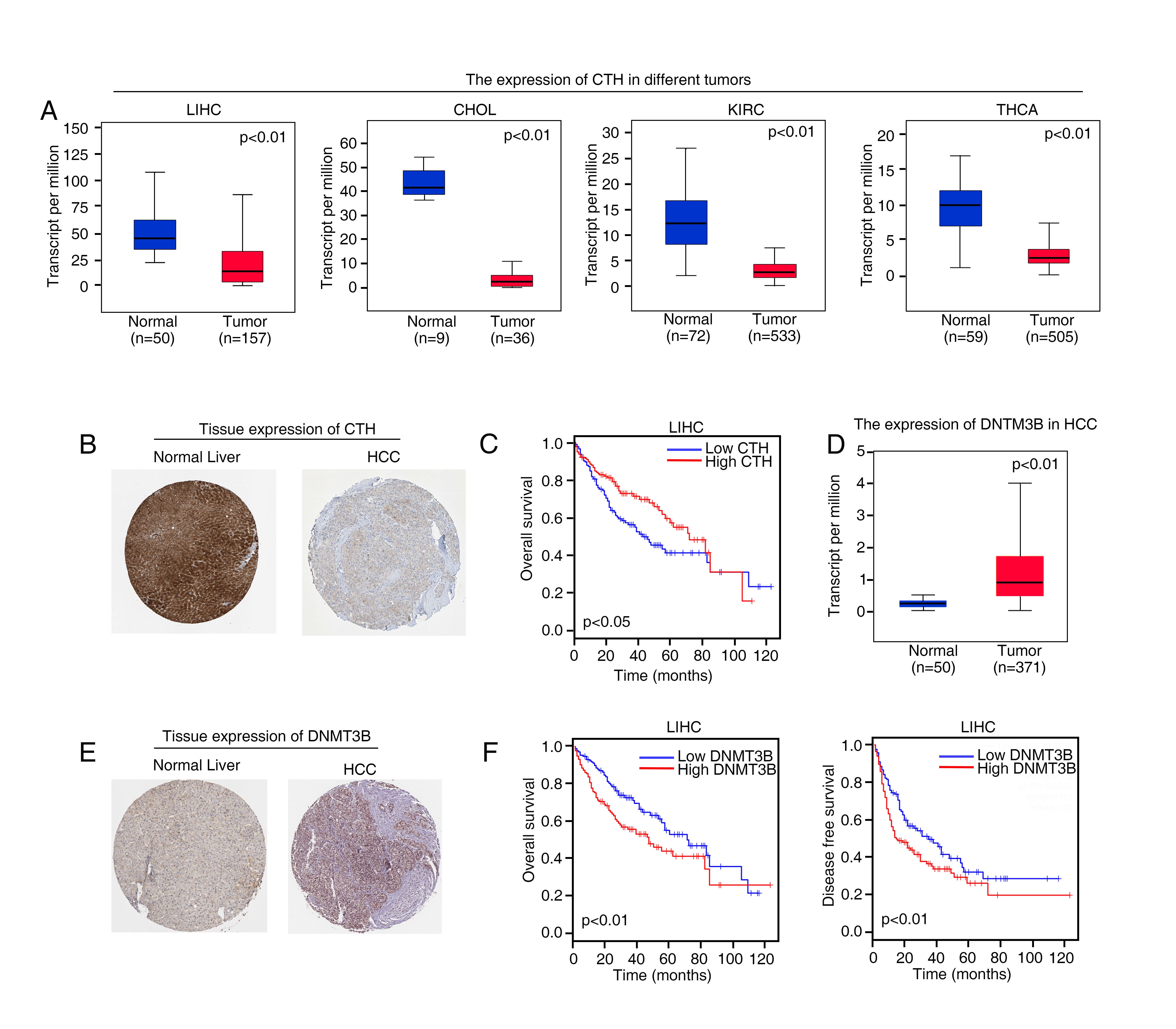

Supplement: Supplementary file 2 — Additional file 2: Supplementary Figure S1, Supplementary Figure S2, Supplementary Figure S3, Supplementary Figure S4, Supplementary Figure S5. [file 13046_2021_1829_MOESM2_ESM.zip › Supplementary Figure S1.jpg]

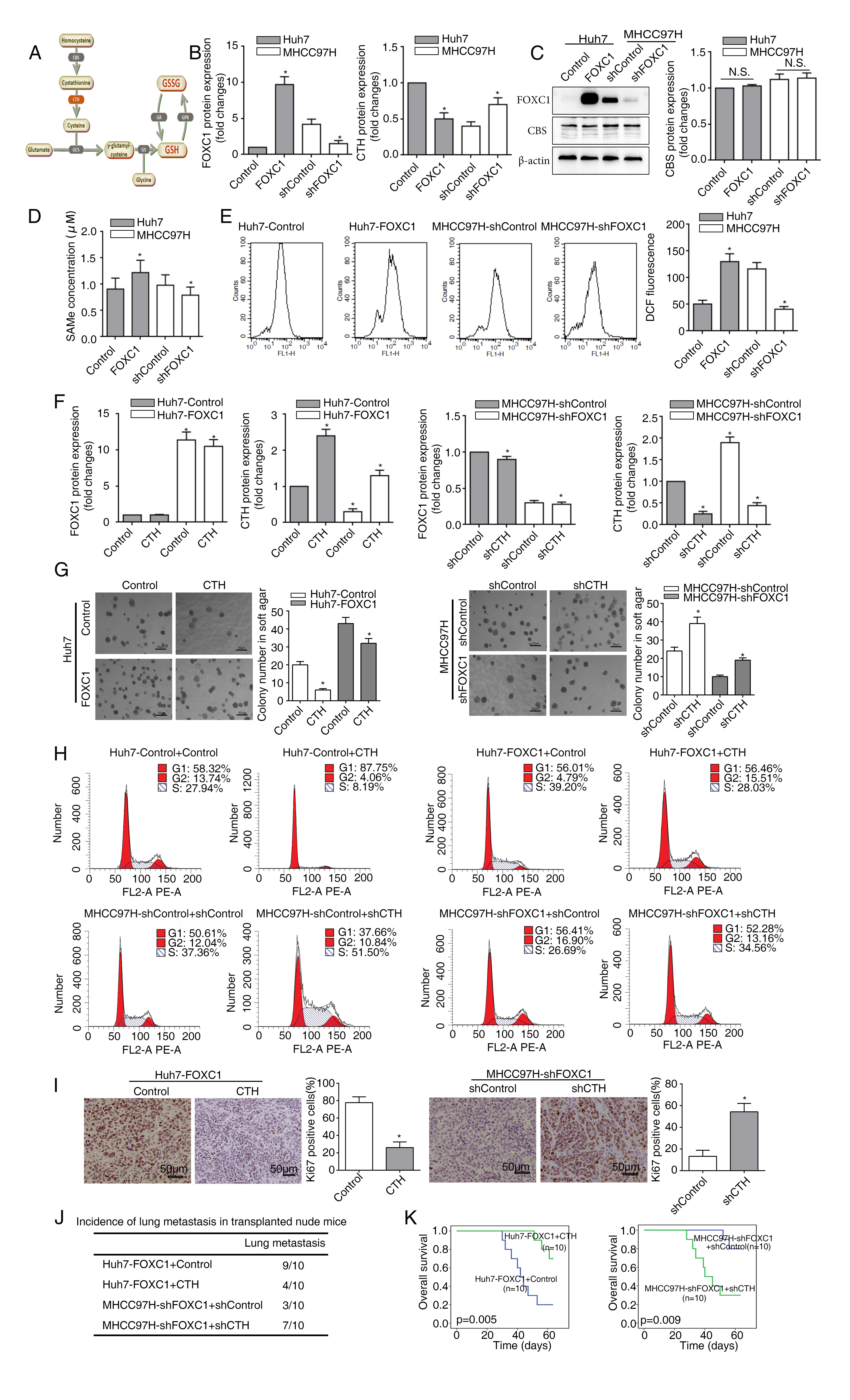

Supplement: Supplementary file 2 — Additional file 2: Supplementary Figure S1, Supplementary Figure S2, Supplementary Figure S3, Supplementary Figure S4, Supplementary Figure S5. [file 13046_2021_1829_MOESM2_ESM.zip › Supplementary Figure S2.jpg]

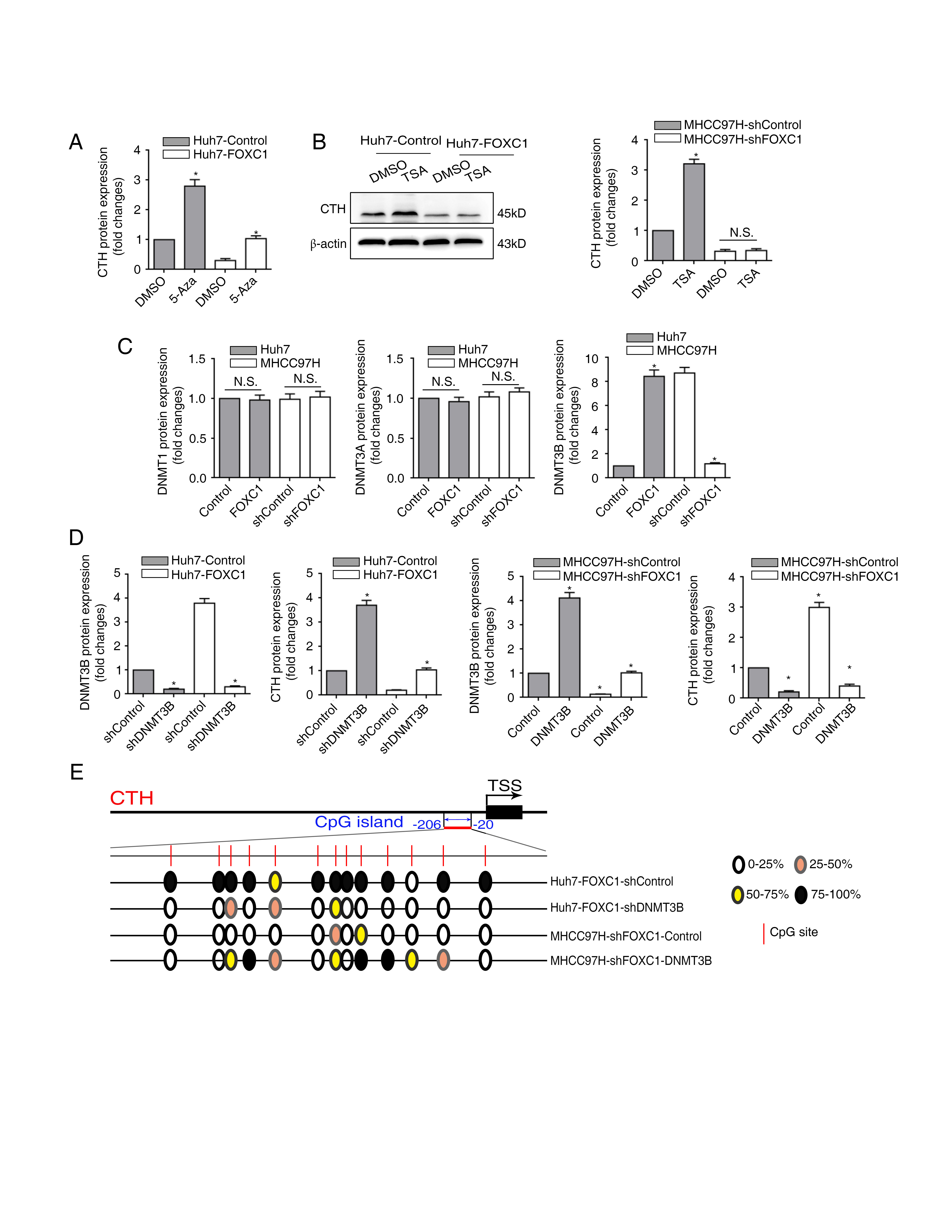

Supplement: Supplementary file 2 — Additional file 2: Supplementary Figure S1, Supplementary Figure S2, Supplementary Figure S3, Supplementary Figure S4, Supplementary Figure S5. [file 13046_2021_1829_MOESM2_ESM.zip › Supplementary Figure S3.jpg]

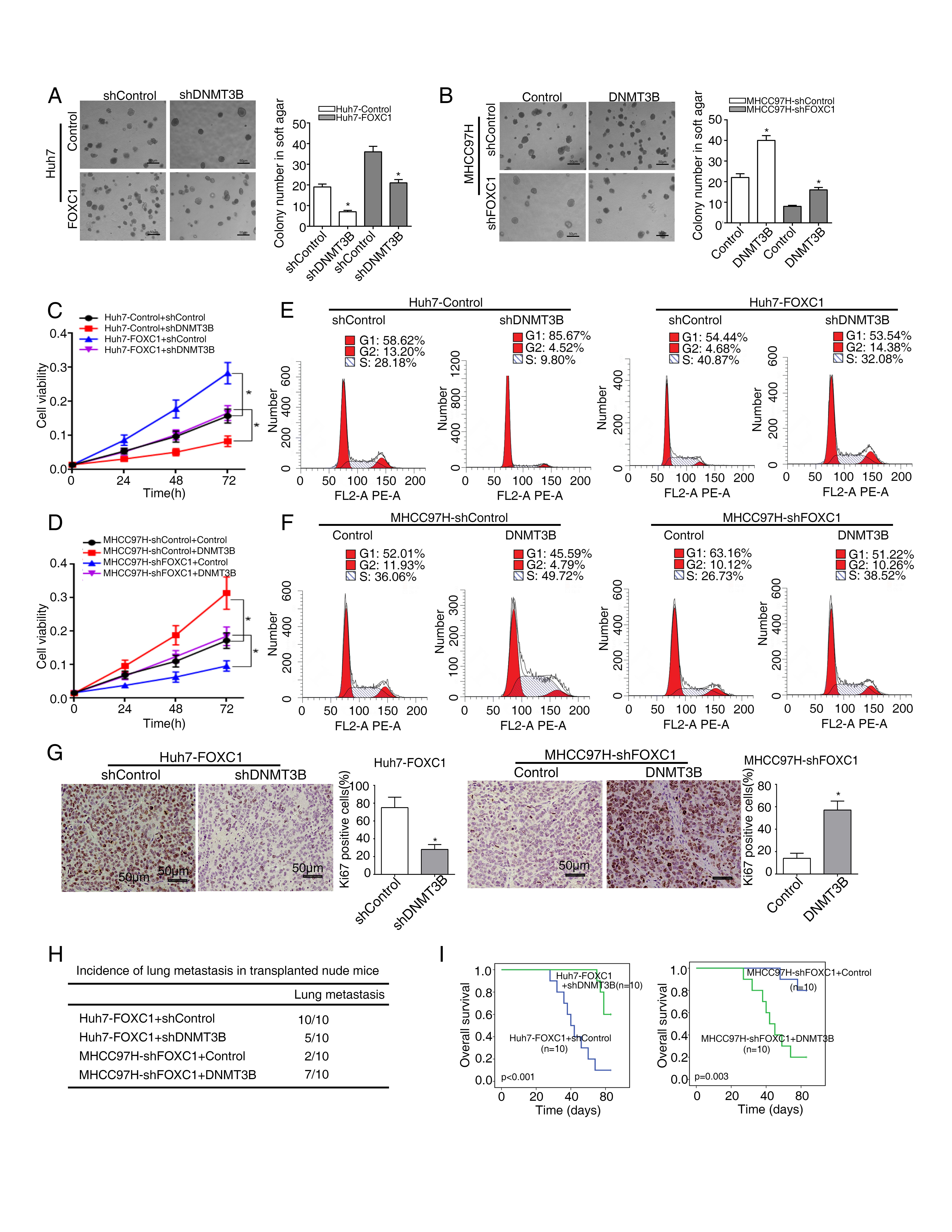

Supplement: Supplementary file 2 — Additional file 2: Supplementary Figure S1, Supplementary Figure S2, Supplementary Figure S3, Supplementary Figure S4, Supplementary Figure S5. [file 13046_2021_1829_MOESM2_ESM.zip › Supplementary Figure S4.jpg]

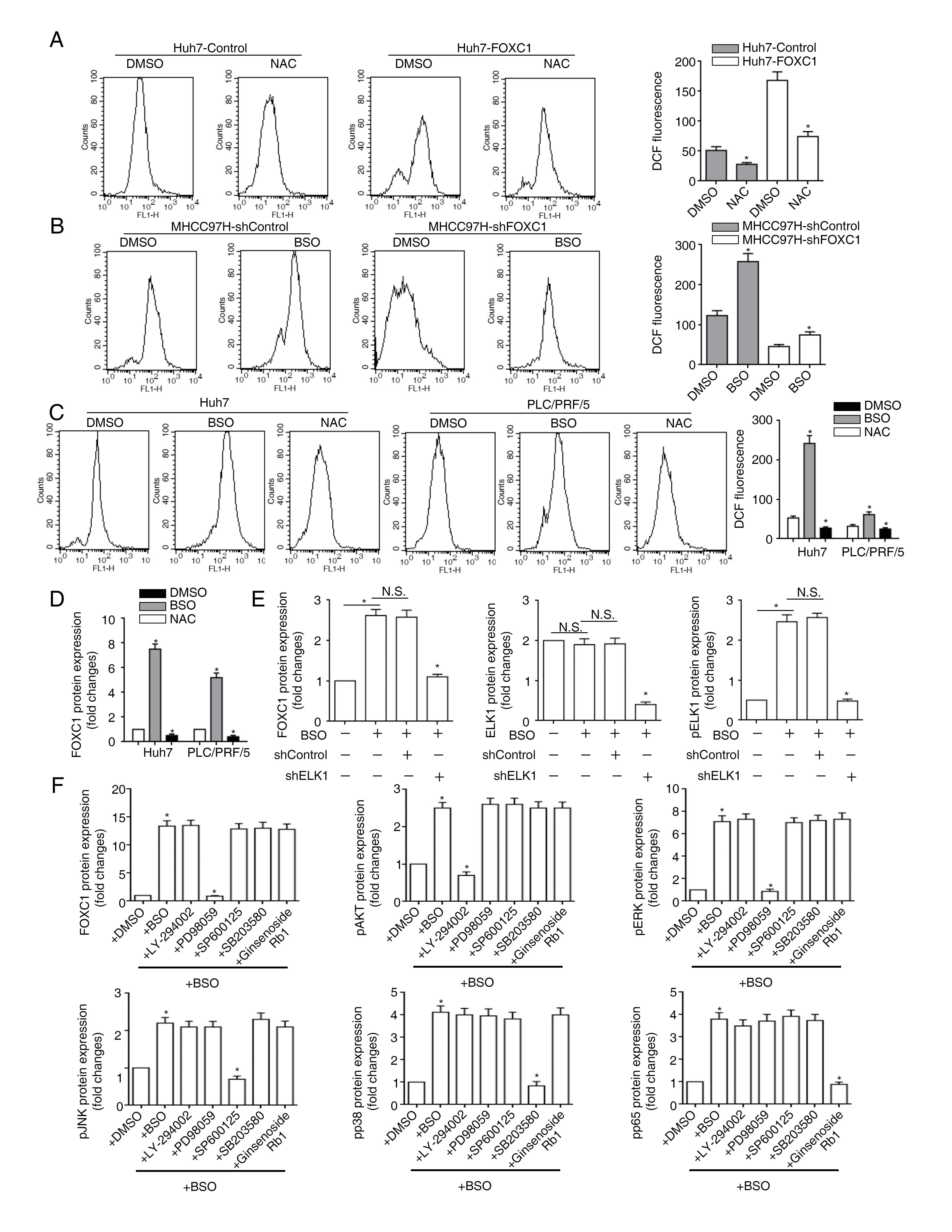

Supplement: Supplementary file 2 — Additional file 2: Supplementary Figure S1, Supplementary Figure S2, Supplementary Figure S3, Supplementary Figure S4, Supplementary Figure S5. [file 13046_2021_1829_MOESM2_ESM.zip › Supplementary Figure S5.jpg]
